# Supplementary material for: Insights into the κ/ι-carrageenan metabolism pathway of some marine Pseudoalteromonas species
Source: Commun Biol. 2019 Dec 19;2:474. doi: 10.1038/s42003-019-0721-y (PMC6923384; doi:10.1038/s42003-019-0721-y)
Supplement: Supplementary file 1 — Supplementary Information [file 42003_2019_721_MOESM1_ESM.pdf]

Supplementary Figures

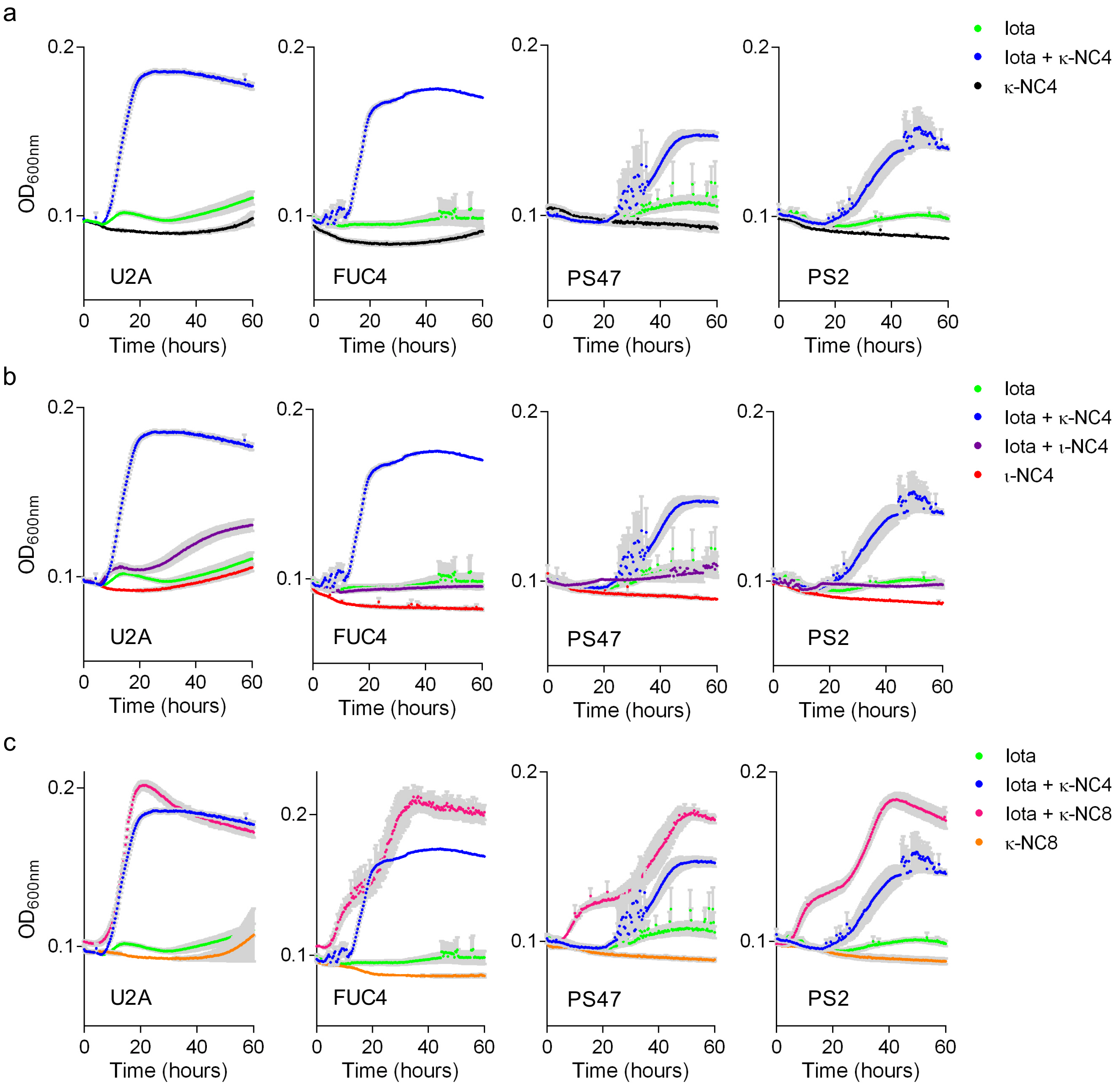

Supplementary Figure 1 (preceding page). Analysis of *Pseudoalteromonas* isolate growth on carrageenan and oligosaccharides. (a) Growth of U2A, FUC4, PS47, and PS2 on 0.4% (w/v) ι-carrageenan (green), 0.4% (w/v) ι-carrageenan with of 0.04% (w/v) κ-NC4 (blue), or 0.4% (w/v) κ-NC4 alone (black). (b) Growth of U2A, FUC4, PS47, and PS2 on 0.4% (w/v) ι-carrageenan (green), 0.4% (w/v) ι-carrageenan with of 0.04% (w/v) κ-NC4 (blue), 0.4% (w/v) ι-carrageenan with of 0.04% (w/v) ι-NC4 (purple), or 0.4% (w/v) ι-NC4 alone (red). (c) Growth of U2A, FUC4, PS47, and PS2 on 0.4% (w/v) ι-carrageenan (green), 0.4% (w/v) ι-carrageenan with of 0.04% (w/v) κ-NC4 (blue), 0.4% (w/v) ι-carrageenan with of 0.04% (w/v) κ-NC8 (pink), or 0.4% (w/v) κ-NC8 alone (orange). Errors bars represent the SEM (*n*=4).

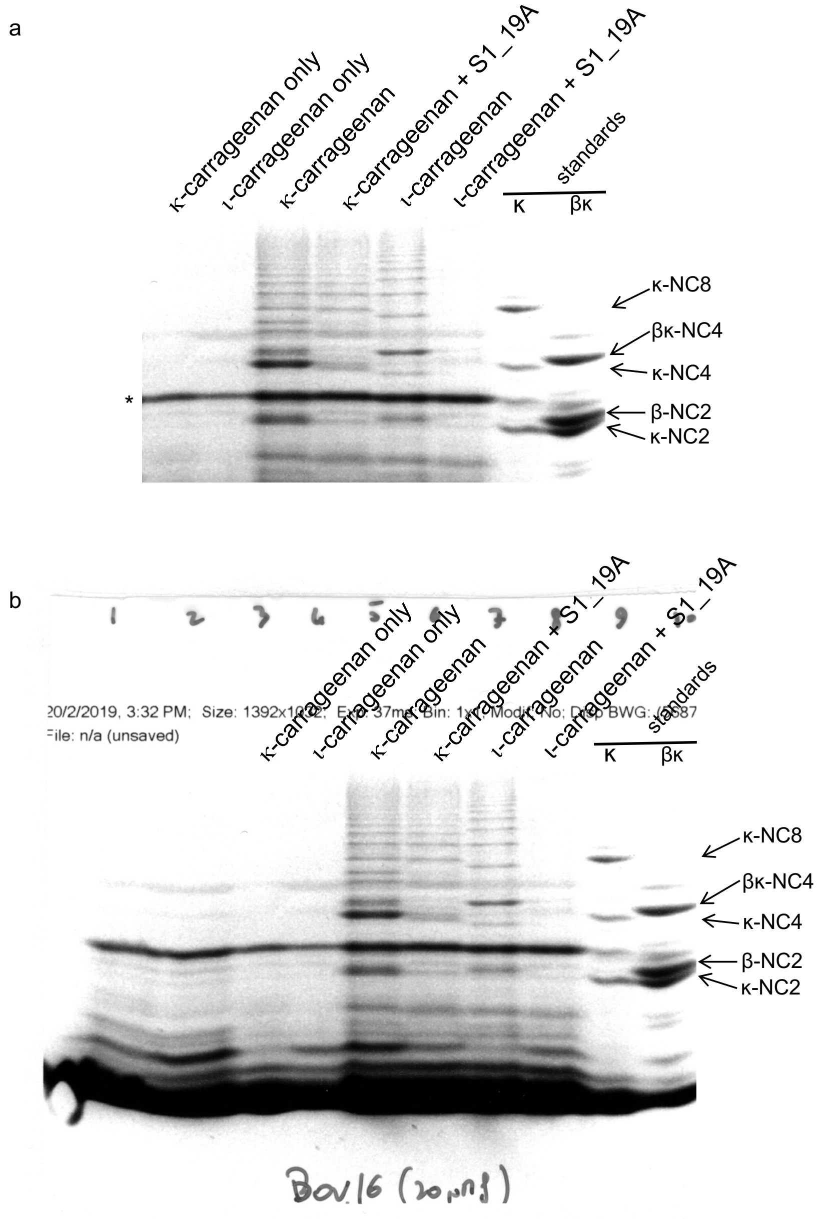

Supplementary Figure 2. Carrageenanase activity of BovGH16. FACE analysis of the products of κ-carrageenan and ι-carrageenan degradation produced by the activity of BovGH16 or BovGH16 added in combination with S1_19A. a) and b) show cropped and uncropped versions of the gel image.

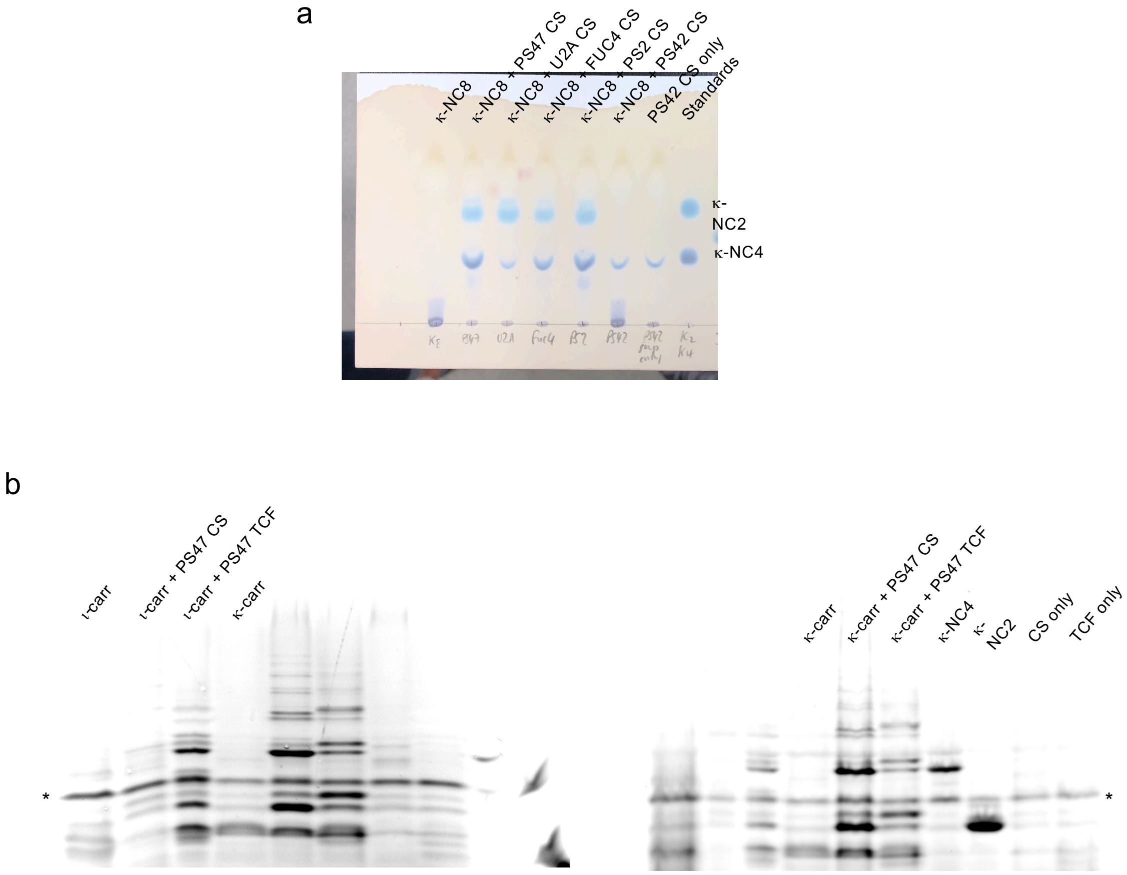

Supplementary Figure 3 (accompanies Figure 2 in the main text). (a) Uncropped image from Figure 2e. Thin layer chromatography (TLC) analysis of κ-NC8 incubated with cell-free culture supernatants (CS) taken from the *Pseudoalteromonas* isolates after growth on MMM supplemented with 0.4% ι-carrageenan and 0.04% κ-NC4 (medium also contained 0.5% galactose for PS42). (b) Uncropped images used to assemble Figure 2f. Fluorophore-assisted carbohydrate electrophoresis (FACE) analysis of ι-carrageenan (ι-carr) and κ-carrageenan (κ-carr) incubated with CS or total cell fraction (TCF) from PS47 after growth on MMM supplemented with 0.4% ι-carrageenan and 0.04% κ-NC4. The asterisks (*) indicate the band corresponding to excess ANTS fluorophore.

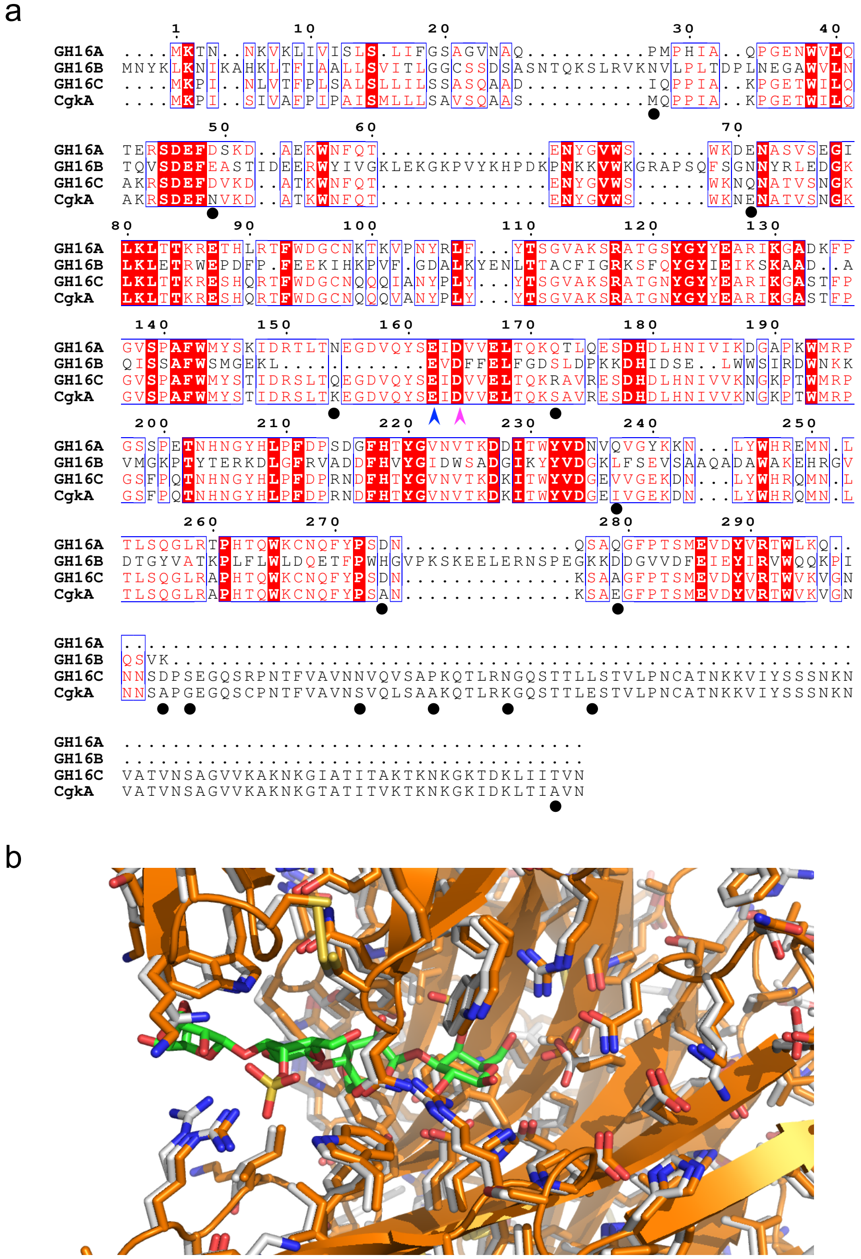

Supplementary Figure 4. Features of the *Pseudoaltermonas* GH16 enzymes. (a) Amino acid sequence alignment of GH16A/B/C with CgkA from *P. carrageenovora* 9^{T}. The catalytic residues are indicated with arrows beneath the sequences. Black circles beneath the sequences indicate the positions that differ between GH16C and CgkA. (b) An overlap of the homology model of GH16A (orange) based on the complex of CgkA with κ-NC4 (grey with κ-NC4 shown as green sticks). The comparison highlights the 100% conservation of active site residues. The active site of GH16C is also identical to CgkA.

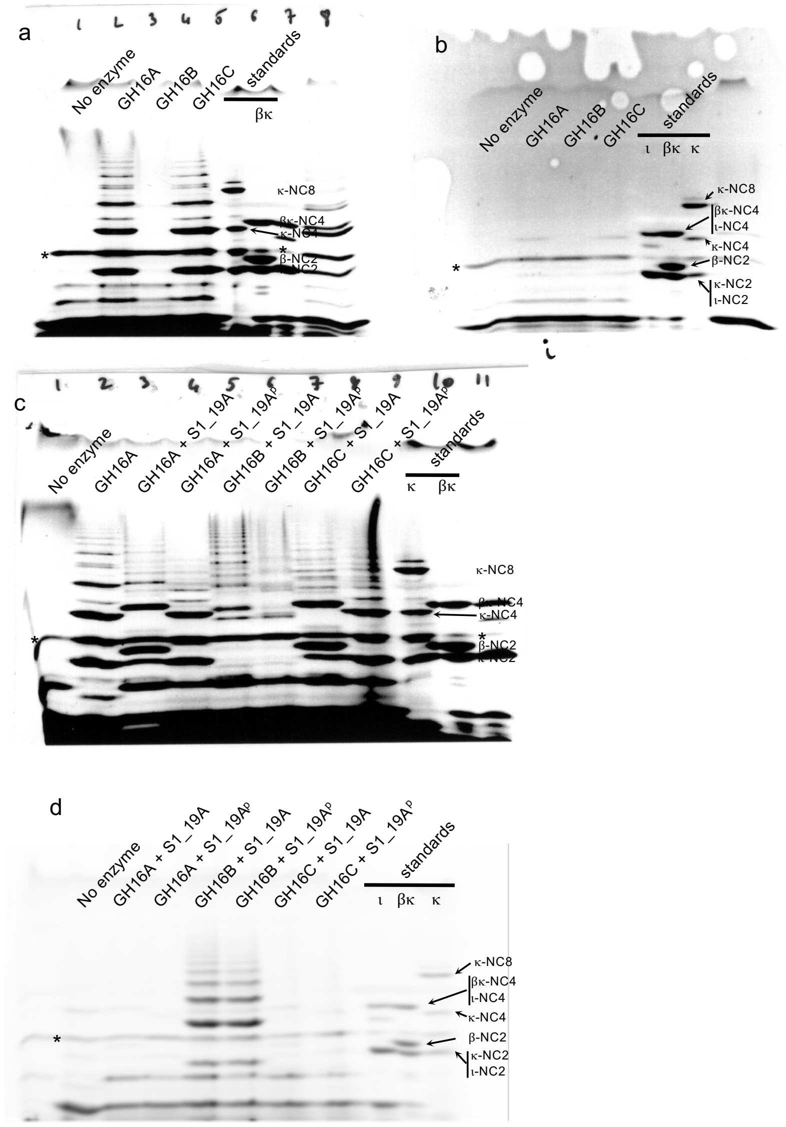

Supplementary Figure 5 (accompanies Figure 3 from main text). The carrageenan degradation properties of recombinant GH16 enzymes from PS47. Uncropped images of gels corresponding to panels a-d from Figure 3. FACE analysis of the products of κ-carrageenan (a) and ι-carrageenan (b) degradation. Panels (c) and (d) show the FACE analysis of κ-carrageenan (c) and ι-carrageenan (d) degradation when the GH16 enzymes are used in conjunction with the S1_19A *endo*-4S-κ/ι-carrageenan sulfatase. The superscript "p" (^{p}) in the sample label indicates that the carrageenan was pretreated overnight with the sulfatase, followed by heat inactivation of the sulfatase then digestion with the GH16. The asterisks (*) indicates the band corresponding to excess ANTS fluorophore.

a

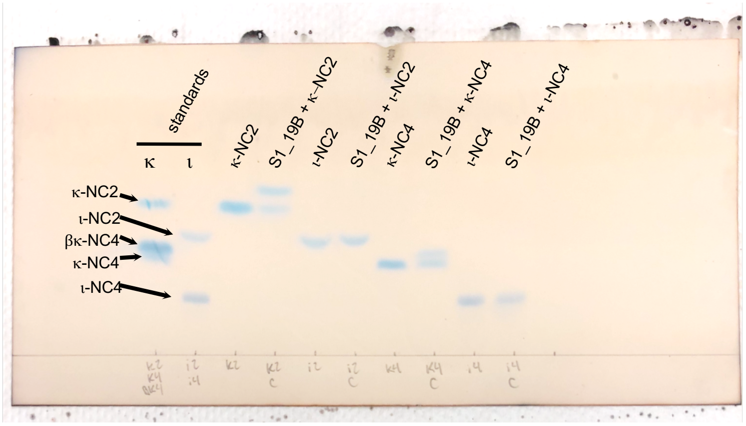

b

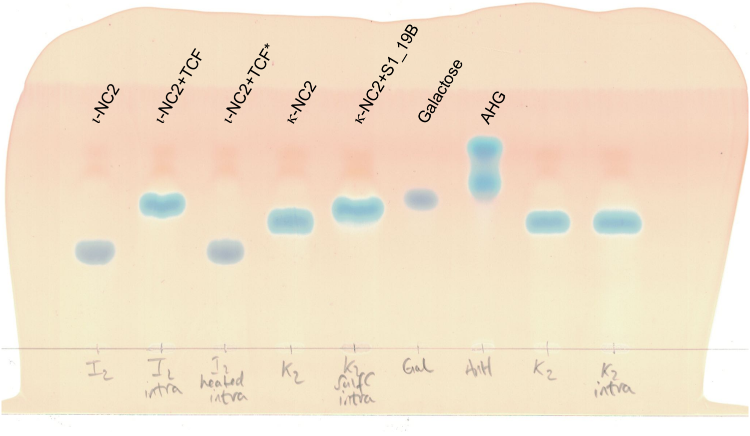

Supplementary Figure 6 (accompanies Figure 4 from the main text). Activity of PS47 sulfatases. (a) Uncropped image of Figure 4a. TLC analysis of S1_19B activity. (b) Uncropped image of Figure 4h. TLC analysis of ι-NC2 conversion by the total cellular fraction (TCF) from PS47. TCF* indicates heat inactivated TCF. κ-NC2 incubated with S1_19B, which produces β-NC2, is shown as a standard.

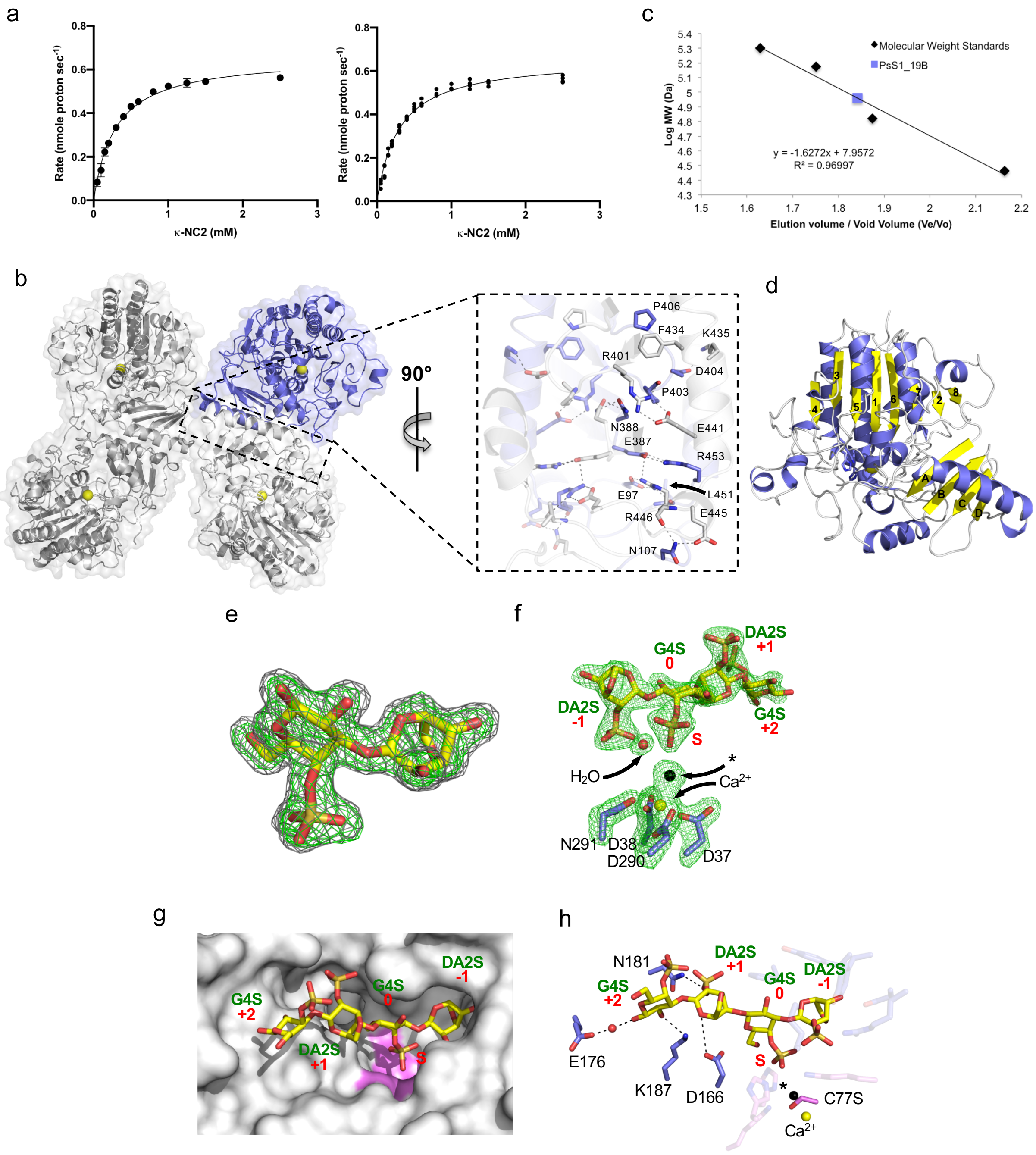

a
Rate (nmole proton sec-1)
κ-NC2 (mM)
c
Molecular Weight Standards
PsS1_19B
y = -1.6272x + 7.9572
R² = 0.96997
Log MW (Da)
Elution volume / Void Volume (Ve/Vo)
b
90°
P406
F434
K435
R401
D404
P403
N388
E441
E387
R453
E97
L451
R446
E445
N107
d
e
f
G4S
0
DA2S
+1
DA2S
-1
G4S
+2
S
H2O
*
Ca2+
N291
D38
D290
D37
g
DA2S
-1
G4S
+2
G4S
0
DA2S
+1
S
h
N181
DA2S
+1
G4S
0
DA2S
-1
G4S
+2
E176
K187
D166
S
*
C77S
Ca2+

Supplementary Figure 7 (preceding page). Activity and structural analysis of S1_19B. (a) Kinetic analysis of S1_19B activity on κ-NC2. Data shown are the mean of four replicates; error bars represent the error. The unaveraged data is shown in the adjacent panel (b) Cartoon representation of the S1_19B tetramer with a monomer colored blue and the remaining monomers colored grey. Calcium ions are shown as yellow spheres. The inset shows the molecular interactions at the dimer interfaces. Amino acid residues are represented as sticks and coloured according to their respective chains; mirrored interactions are shown as transparent amino acid residues, and hydrogen bonds are represented as dashed lines. (c) Oligomeric state determination of S1_19B based on elution volume when compared to the calibration proteins ranging from 29 kDa to 200 kDa. S1_19B is represented by a purple square. (d) Cartoon representation of S1_19B revealing the two subdomains of mixed α/β topology (α-helices coloured slate, β-sheets coloured yellow). N-terminal subdomain meandering β-sheets are numbered 1-8 and the C-terminal subdomain anti-parallel β-sheets are lettered A-D. Residues comprising the S-subsite are represented as slate sticks and the calcium ion as a yellow sphere. (e) Representative electron density for κ-NC2 modeled into the active site. The gray mesh shows the electron density map as a maximum likelihood/σ_a-weighted $2F_o$-F_c map contoured at 1.0 σ. The green mesh shows the electron density map as a maximum likelihood/σ_a-weighted F_o-F_c map (contoured at 3.0 σ) produced by refinement with the κ-NC2 atoms omitted. (f) Representative electron densities for ι-NC4, calcium ion, calcium binding residues, and the counter ion modeled into the active site. The green mesh shows the electron density map as a maximum likelihood/σ_a-weighted F_o-F_c map (contoured at 3.0 σ) produced by refinement with the corresponding residues omitted. (g) Close up of S1_19B C77S active site pocket with bound ι-NC4. The solvent accessible surface is coloured in gray with the area comprising the S-subsite coloured in violet. (h) The ι-NC4 bound in the active site with a focus on the DA2S and G4S residues in the potential +1 and +2 subsites, respectively.

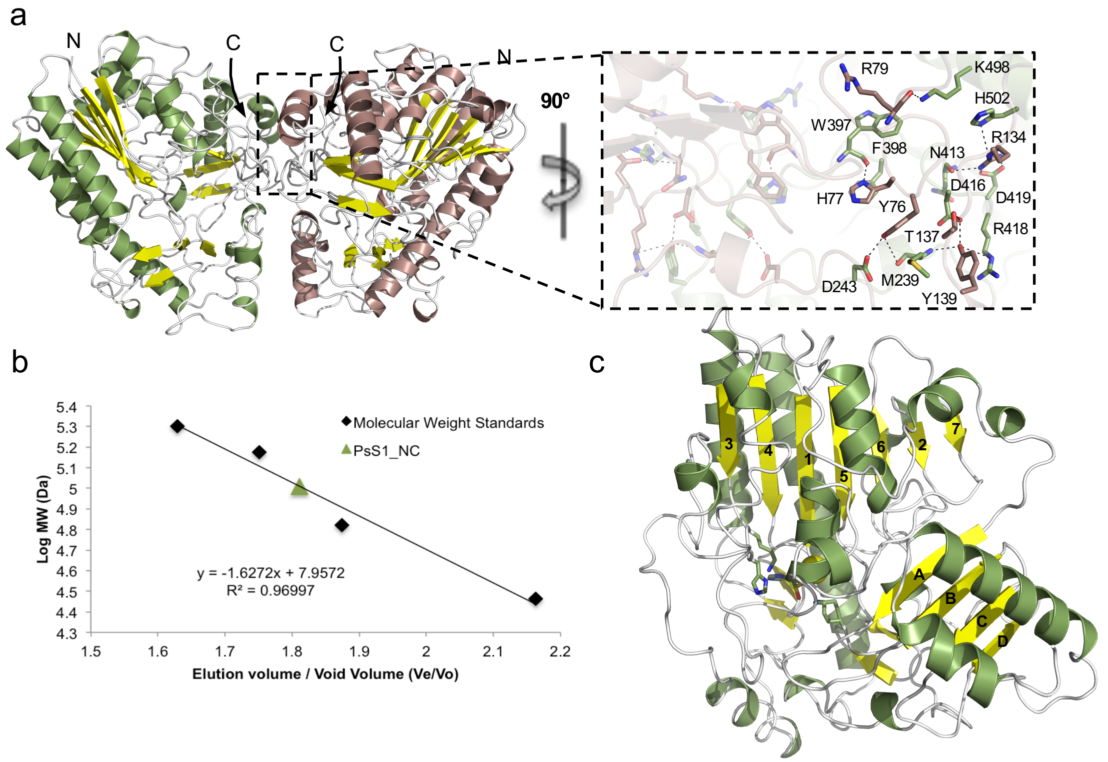

Supplementary Figure 8. Structural analysis of S1_NC. (a) Cartoon representation of the S1_NC dimer. The α-helices are coloured according to either chain A or chain B as green or brown, respectively. β-sheets are coloured yellow and polypeptide termini are labeled (N) or (C). The inset shows the molecular interactions at the dimer interface. Amino acid residues are represented as sticks and coloured according to their respective chains; mirrored interactions are shown as transparent amino acid residues and hydrogen bonds are represented as dashed lines. (b) Oligomeric state determination of S1_NC based on elution volume when compared to the calibration proteins ranging from 29 kDa to 200 kDa. S1_NC is represented as a green triangle. (c) Cartoon representation of the S1_NC is characterized by two subdomains of mixed α/β topology (α-helices coloured green, β-sheets coloured yellow). N-terminal subdomain meandering β-sheets are numbered 1-7 and the C-terminal subdomain anti-parallel β-sheets are lettered A-D. Residues comprising the S-subsite are represented as green sticks and the calcium ion as a yellow sphere.

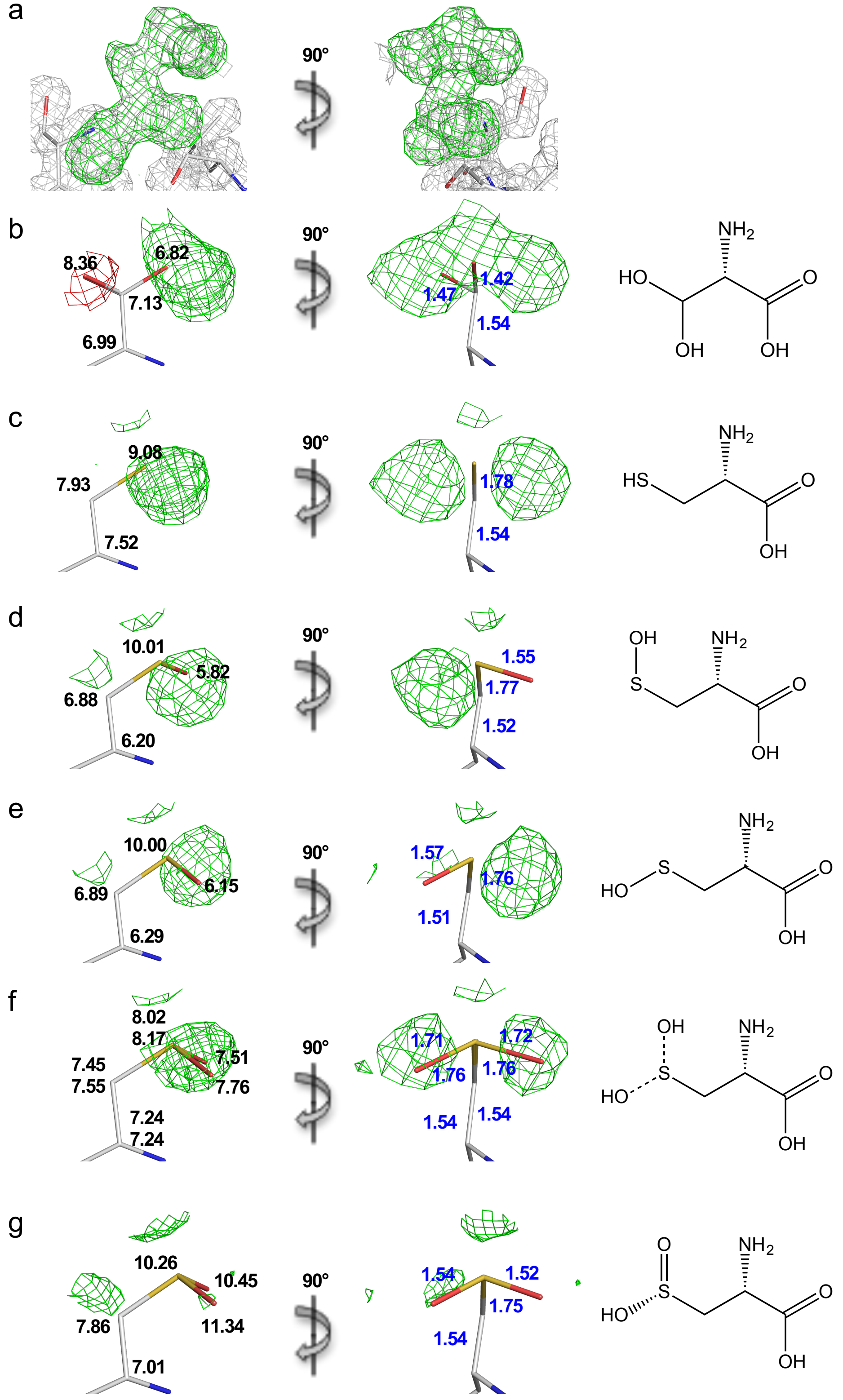

a
90°
b
8.36
6.82
7.13
6.99
1.47
1.42
1.54
NH2
HO
O
OH
OH
c
9.08
7.93
7.52
1.78
1.54
HS
d
10.01
5.82
6.88
6.20
1.55
1.77
1.52
S
e
10.00
6.15
6.89
6.29
1.57
1.76
1.51
f
8.02
8.17
7.45
7.55
7.51
7.76
7.24
7.24
1.71
1.72
1.76
1.76
1.54
1.54
g
10.26
10.45
7.86
11.34
7.01
1.54
1.52
1.75
1.54

Supplementary Figure 9 (preceding page). X-ray crystallographic analysis of the catalytic residue in S1_NC to reveal its post-translational modification. (a) Representative electron density for S1_NC active site residues. The gray mesh shows the electron density map as a maximum likelihood/σ_a-weighted $2F_o$-F_c map contoured at 1.0 σ. The green mesh shows the unbiased electron density map as a maximum likelihood/σ_a-weighted F_o-F_c map (contoured at 3.0 σ) produced by refinement with the atoms for residue 84 omitted. Residues are coloured as gray lines. In panels b – g, representative electron density for S1_NC residue 84 is shown when modeled as FGly (b), cysteine (c), cysteinsulfenic acid conformation-A (d), cysteinsulfenic acid conformation-B (e), cysteinsulfenic acid in both conformations each having 50 % occupancy (f), and cysteinsulfinic acid (g). The green mesh shows the electron density map as a maximum likelihood/σ_a-weighted F_o-F_c map (contoured at 3.0 σ) produced by refinement with the residue atoms present. All colouring for panels b-g are the same as in (a). Respective B-factors and bond lengths are labeled in black and blue, respectively.

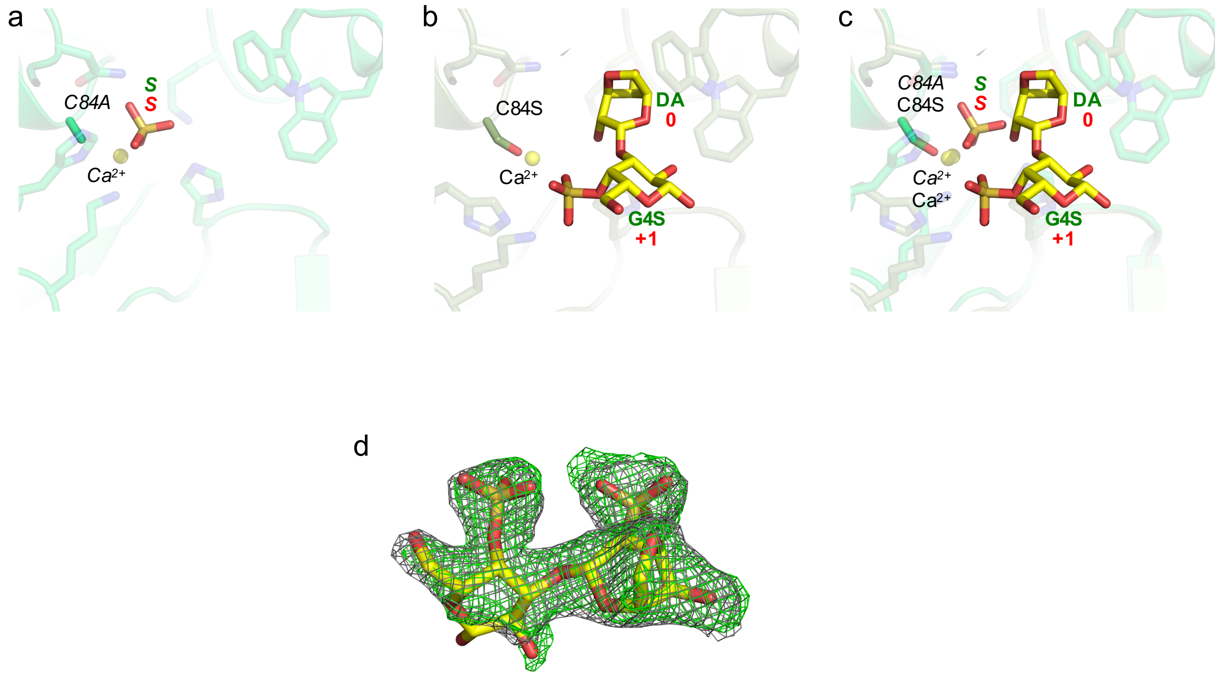

Supplementary Figure 10. Sulfate, κ-NC2, and ι-NC4 complexes of S1_NC. (a) The active site of S1_NC C84A in complex with sulfate. (b) The active site of S1_NC C84S in complex with κ-NC2. (c) An overlay of the sulfate and κ-NC2 complexes. (d) Representative electron density for ι-NC4 modeled into the active site; density was only evident for two of the sugar residue ultimately leading to the sugar being modeled as ι-NC2. The gray mesh shows the electron density map as a maximum likelihood/σ_a-weighted $2F_o$-F_c map contoured at 1.0 σ. The green mesh shows the electron density map as a maximum likelihood/σ_a-weighted F_o-F_c map (contoured at 3.0 σ) produced by refinement with the ι-NC4 atoms omitted.

a

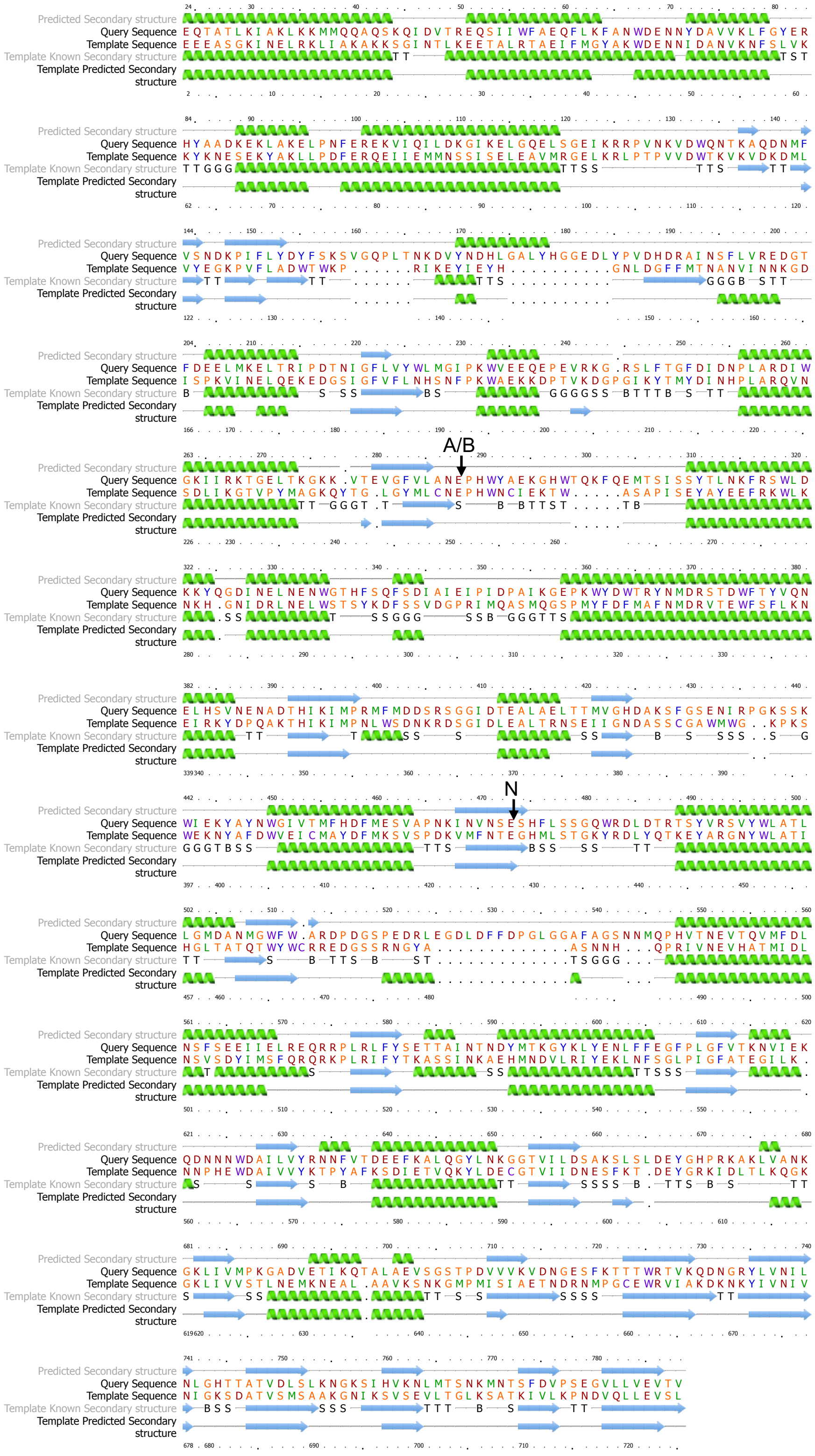

Query = PS47 GH167

Template = BovGH167 Structure (PDB ID 6PTM)

b

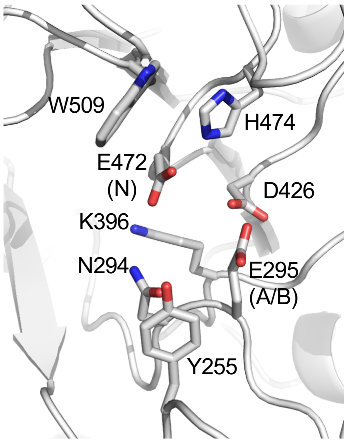

W509
H474
E472
(N)
D426
K396
N294
E295
(A/B)
Y255

c

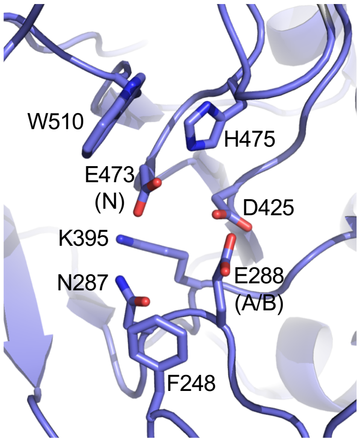

W510
H475
E473
(N)
D425
K395
N287
E288
(A/B)
F248

d

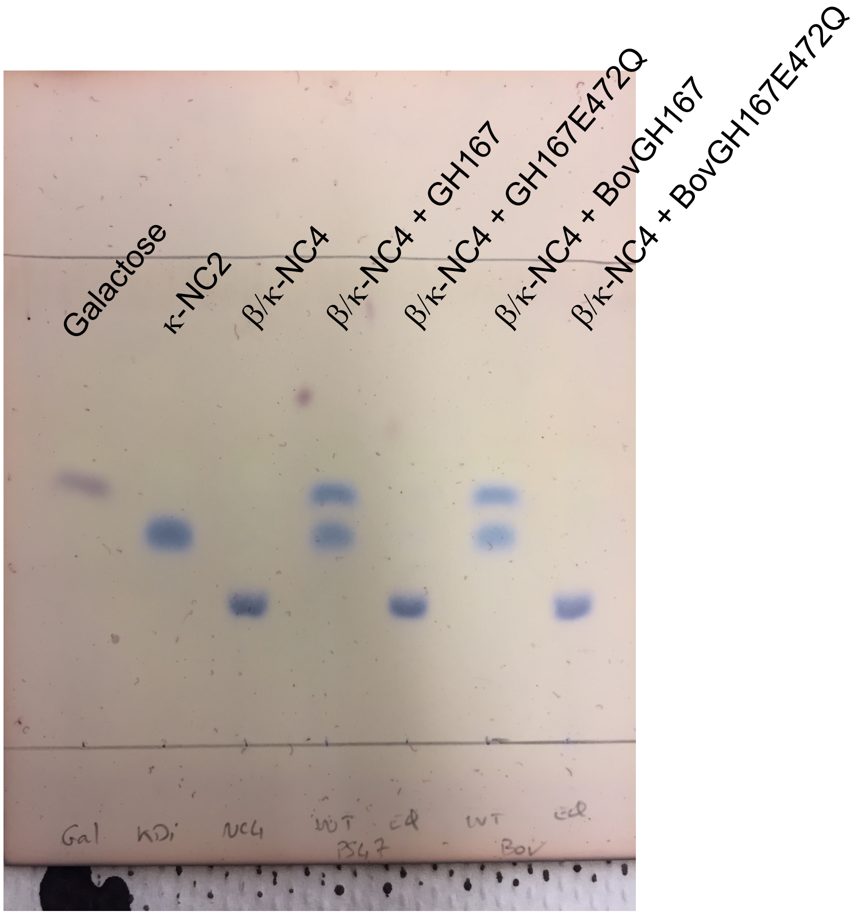

Galactose
κ-NC2
β/κ-NC4
β/κ-NC4 + GH167
β/κ-NC4 + GH167E472Q
β/κ-NC4 + BovGH167
β/κ-NC4 + BovGH167E472Q

Supplementary Figure 11 (preceding 2 pages). Analysis of GH167 and BovGH167. (a) Amino acid sequence alignment of GH167 (query) with BovGH167 (template) generated by one-to-one threading with Phyre2. The predicted secondary structure is show for both sequences as well the known secondary structure for BovGH167; α-helices are shown in green and β-strands as blue arrows. The overall amino acid sequence identity is 36%. (b) Amino acid composition in the putative -1 subsite of BovGH167. (c) Amino acid composition of the putative -1 subsite in GH167 using the model of GH167 generated with Phyre2 one-to-one threading and the structure of BovGH167 as a template. In panels a-c the nucleophile is indicated by an N and the acid-base as A/B. The overall identity of the -1 subsites reflects the similar specificities of the two enzymes. (d) TLC analysis of the nucleophile mutants of GH167 and BovGH167.

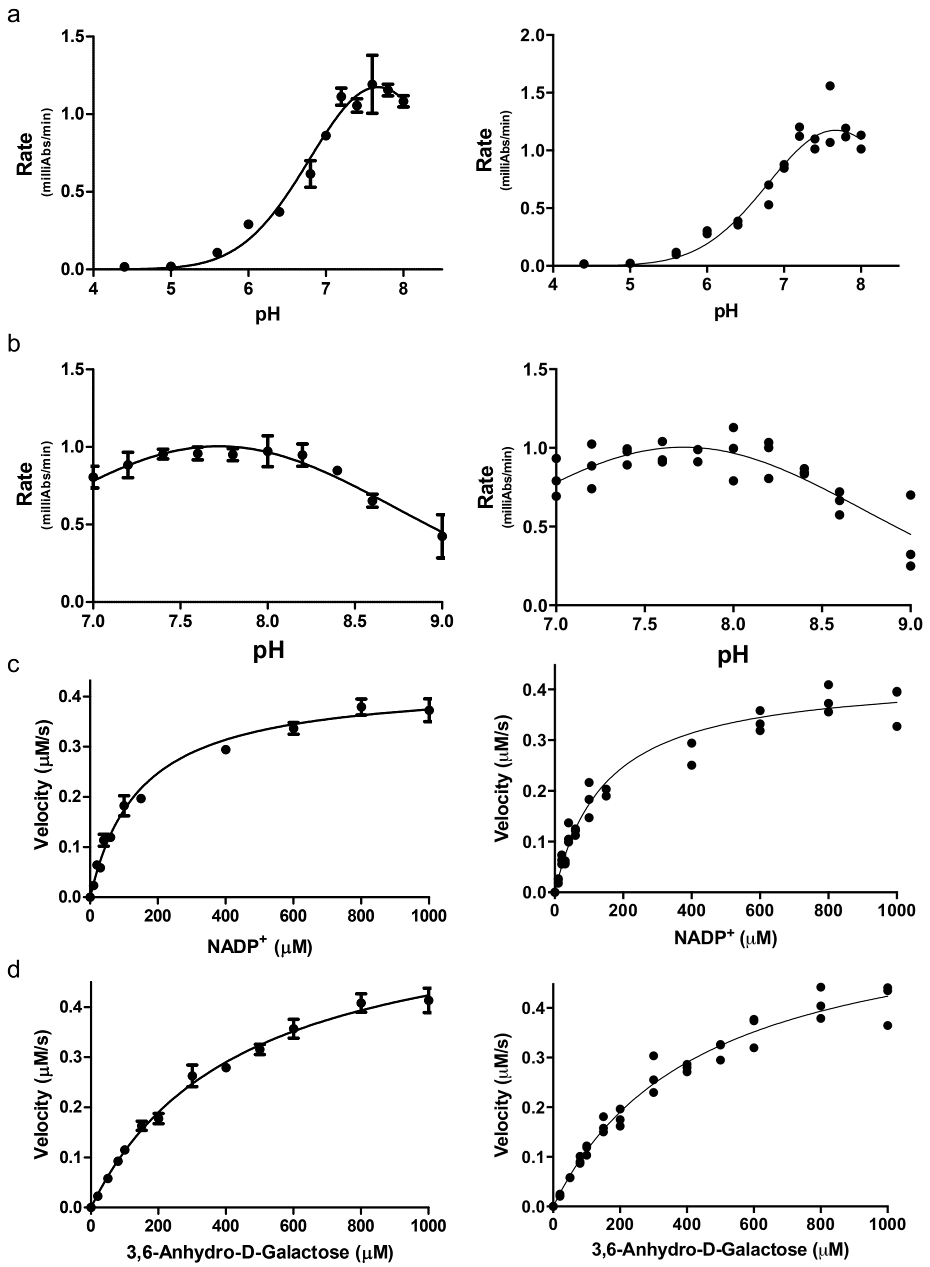

Supplementary Figure 12. Activity analysis of DauA. (a) DauA rate (arbitrary units) plotted vs pH (McIlvaine's buffer). (b) DauA rate (arbitrary units) plotted vs pH (Tris-HCl buffer). Michaelis-Menten plots of rate vs (c) NADP+ and (d) 3,6-anhydro-D-galactose performed at 25 ºC in 20 mM Tris-HCl (pH 8.0) with 0.5 M NaCl and 20 nM of enzyme. Left panels show averaged data (duplicate or triplicate measurements) with error bars showing standard deviations and right panels show unaveraged data.

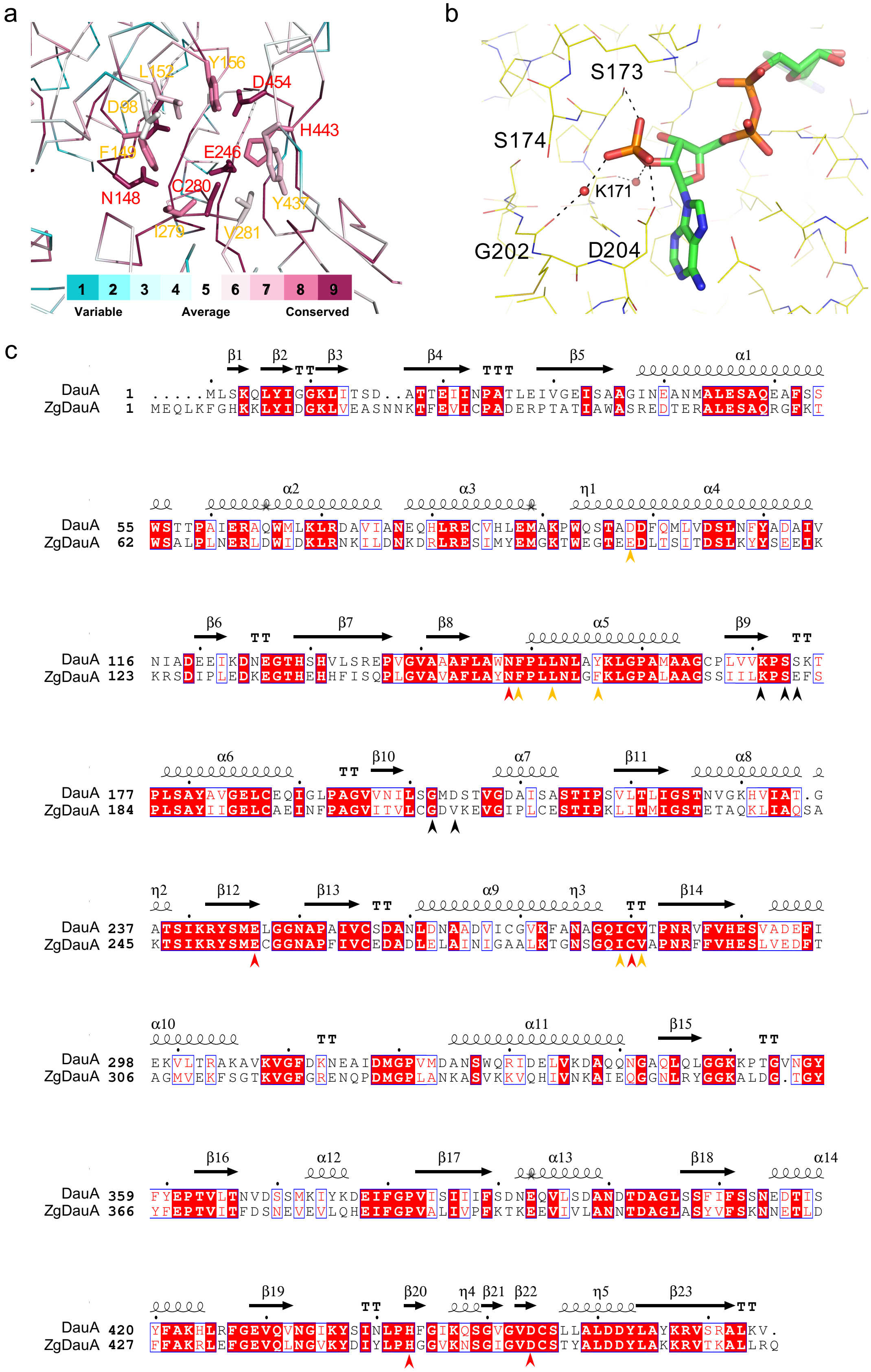
a
L152
Y156
D454
D98
H443
F149
E246
C280
N148
Y437
I279
V281
1 2 3 4 5 6 7 8 9
Variable
Average
Conserved
b
S173
S174
K171
G202
D204
c
β1 β2 β3 β4 β5 α1
DauA 1 .....MLSKQLYIGGKLITSD..ATTEIINPATLEIVGEISAAGINEANMALESAQEAFSS
ZgDauA 1 MEQLKFGHKKLYIDGKLVEASNNKTFEVICPADERPTATIAWASREDTERALESAQRGFKT
α2 α3 η1 α4
DauA 55 WSTTPAIERAQWMLKLRDAVIANEQHLRECVHLEMAKPWQSTADDFQMLVDSLNFYADAIV
ZgDauA 62 WSALPLNERLDWIDKLRNKILDNKDRLRESIMYEMGKTWEGTEEDLTSITDSLKYYSEEIK
β6 β7 β8 α5 β9
DauA 116 NIADEEIKDNEGTHSHVLSREPVGVAAAFLAWNFPLLNLAYKLGPAMAAGCPLVVKPSSKT
ZgDauA 123 KRSDIPLEDKEGTHEHHFISQPLGVAVAFLAYNFPLLNLGFKLGPALAAGSSIILKPSEFS
α6 β10 α7 β11 α8
DauA 177 PLSAYAVGELCEQIGLPAGVVNILSGMDSTVGDAISASTIPSVLTLIGSTNVGKHVIAT.G
ZgDauA 184 PLSAYIIGELCAEINFPAGVITVLCGDVKEVGIPLCESTIPKLITMIGSTETAQKLIAQSA
η2 β12 β13 α9 η3 β14
DauA 237 ATSIKRYSMELGGNAPAIVCSDANLDNAADVICGVKFANAGQICVTPNRVFVHESVADEFI
ZgDauA 245 KTSIKRYSMECGGNAPFIVCEDADLELAINIGAALKTGNSGQICVAPNRFFVHESLVEDFT
α10 α11 β15
DauA 298 EKVLTRAKAVKVGFDKNEAIDMGPVMDANSWQRIDELVKDAQQNGAQLQLGGKKPTGVNGY
ZgDauA 306 AGMVEKFSGTKVGFGRENQPDMGPLANKASVKKVQHIVNKAIEQGGNLRYGGKALDG.TGY
β16 α12 β17 α13 β18 α14
DauA 359 FYEPTVLTNVDSSMKIYKDEIFGPVISIIIFSDNEQVLSDANDTDAGLSSFIFSSNEDTIS
ZgDauA 366 YFEPTVITFDSNEVEVLQHEIFGPVALIVPFKTKEEVIVLANNTDAGLASYVFSKNNETLD
β19 β20 η4 β21 β22 η5 β23
DauA 420 YFAKHLRFGEVQVNGIKYSINLPHFGIKQSGVGVDCSLLALDDYLAYKRVSRALKV.
ZgDauA 427 FFAKRLEFGEVQLNGVKYDIYLPHGGVKNSGIGVDCSTYALDDYLAKKRVTKALLRQ

Supplementary Figure 13 (preceding page). Structural features of DauA. (a) Consurf analysis of the DauA active site based on homologs greater than 35% amino acid sequence identity. Residues likely involved in catalysis (labeled in red) and substrate binding (labeled in yellow) are shown as sticks. (b) Recognition of the sulfate group distinguishing $NADP^+$ (shown as sticks) from NAD^+. Hydrogen bonds are indicated as dashed lines. (c) Amino acid sequence alignment with DauA from *Z. galactanivorans* (ZgDauA). Likely catalytic residues are indicated by red arrows, substrate (AHG) binding residues by yellow arrows, and residues recognizing the sulfate group of $NADP^+$ by black arrows.

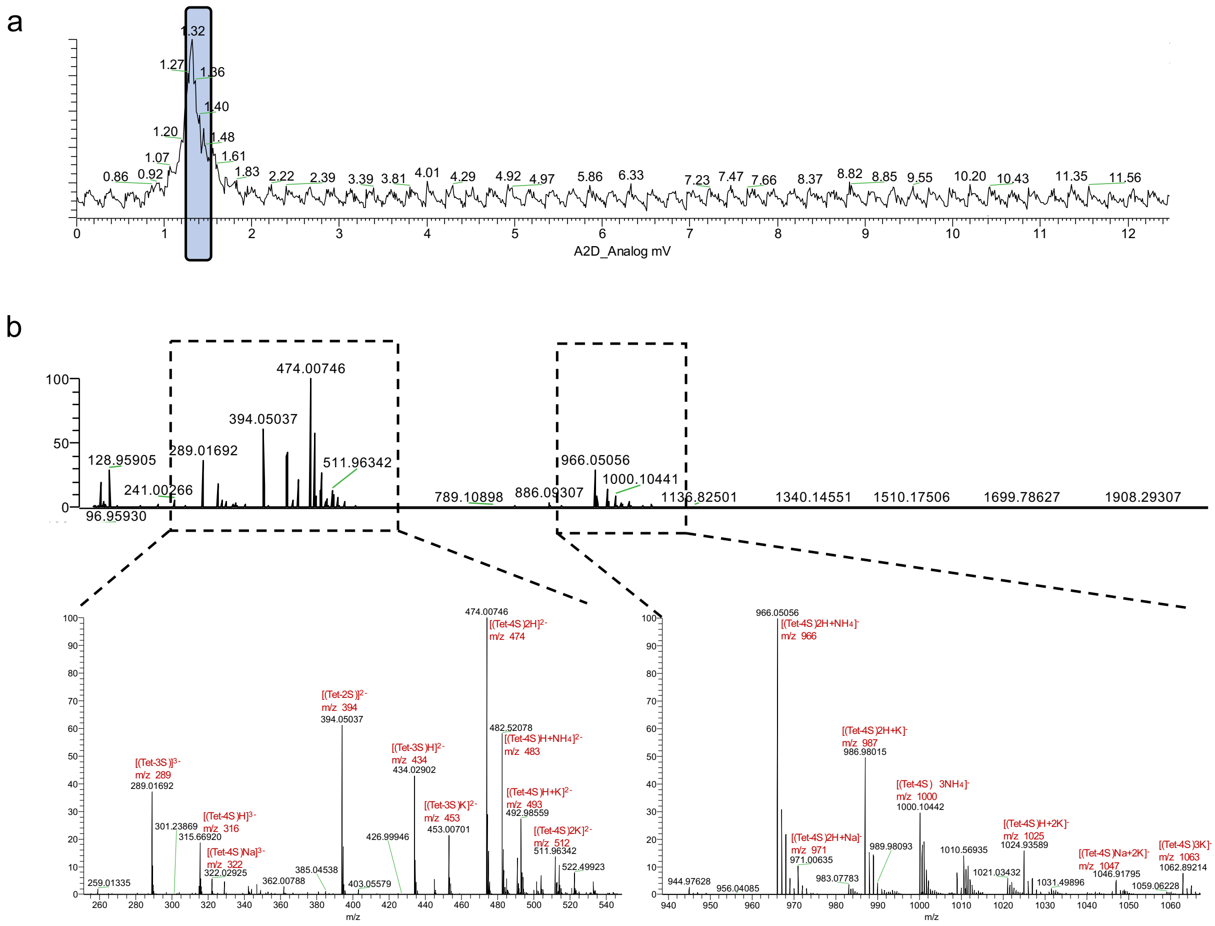
a
A2D_Analog mV
b
474.00746
394.05037
289.01692
128.95905
511.96342
241.00266
96.95930
789.10898
886.09307
966.05056
1000.10441
1136.82501
1340.14551
1510.17506
1699.78627
1908.29307
[(Tet-3S)]3-
m/z 289
[(Tet-4S)H]3-
m/z 316
[(Tet-4S)Na]3-
m/z 322
[(Tet-2S)]2-
m/z 394
[(Tet-3S)H]2-
m/z 434
[(Tet-3S)K]2-
m/z 453
[(Tet-4S)2H]2-
m/z 474
[(Tet-4S)H+NH4]2-
m/z 483
[(Tet-4S)H+K]2-
m/z 493
[(Tet-4S)2K]2-
m/z 512
m/z
[(Tet-4S)2H+NH4]-
m/z 966
[(Tet-4S)2H+Na]-
m/z 971
[(Tet-4S)2H+K]-
m/z 987
[(Tet-4S) 3NH4]-
m/z 1000
[(Tet-4S)H+2K]-
m/z 1025
[(Tet-4S)Na+2K]-
m/z 1047
[(Tet-4S)3K]-
m/z 1063
m/z

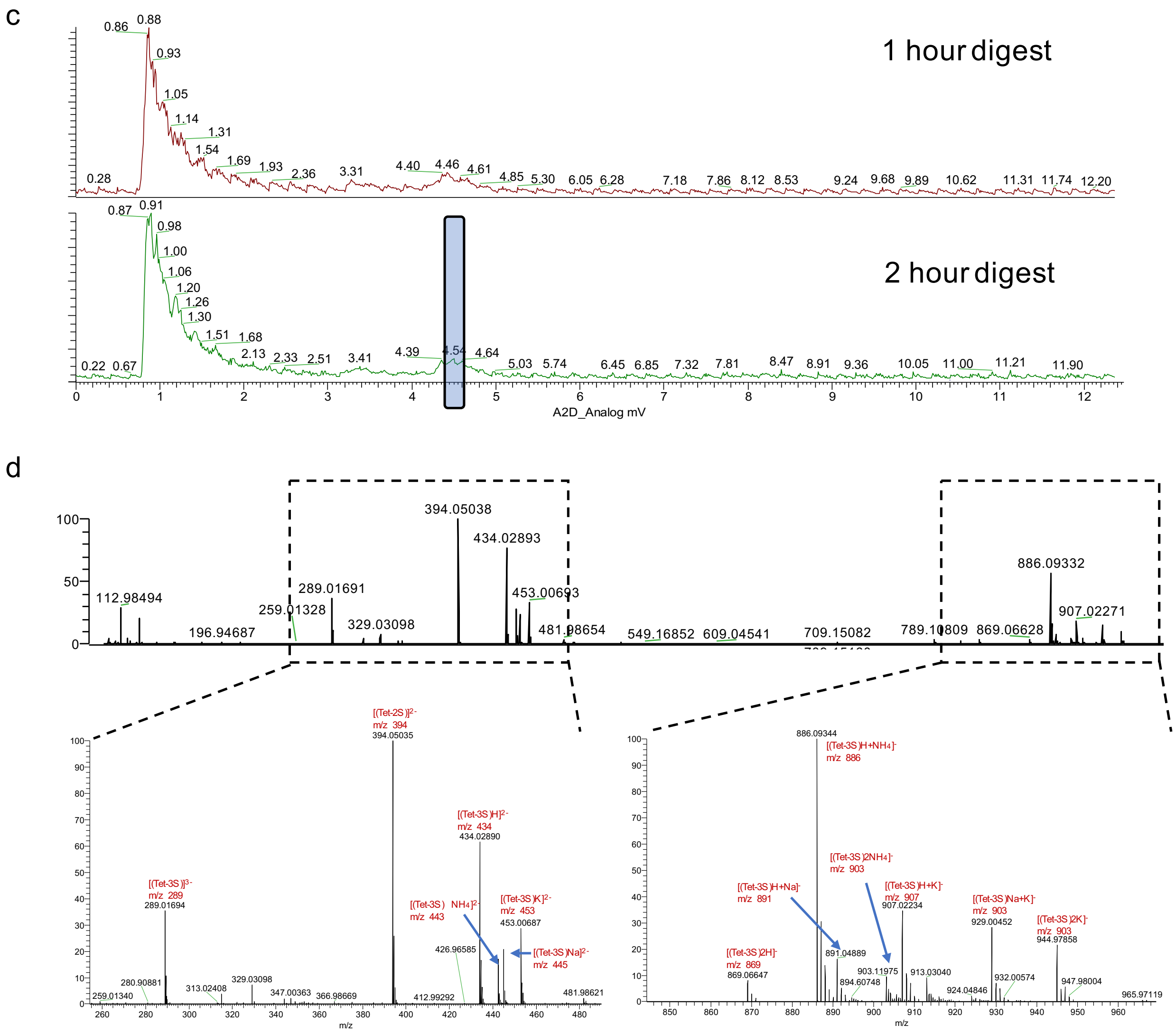

Supplementary Figure 14 (and preceding page). Mass spectrometry analysis of S1_19A activity on ι-NC4. (a) LC-MS total ion chromatograms of ι-NC4 and (b) HRMS spectrum of the peak highlighted in panel (a) with specific regions expanded and annotated. (c) LC-MS total ion chromatograms of ι-NC4 treated for 1 (top) and 2 (bottom) hours with S1_19A. (d) the HRMS spectrum of the peak highlighted in panel (d) with specific regions expanded and annotated. See Supplementary Table 7 for a list of expected and measured species.

a

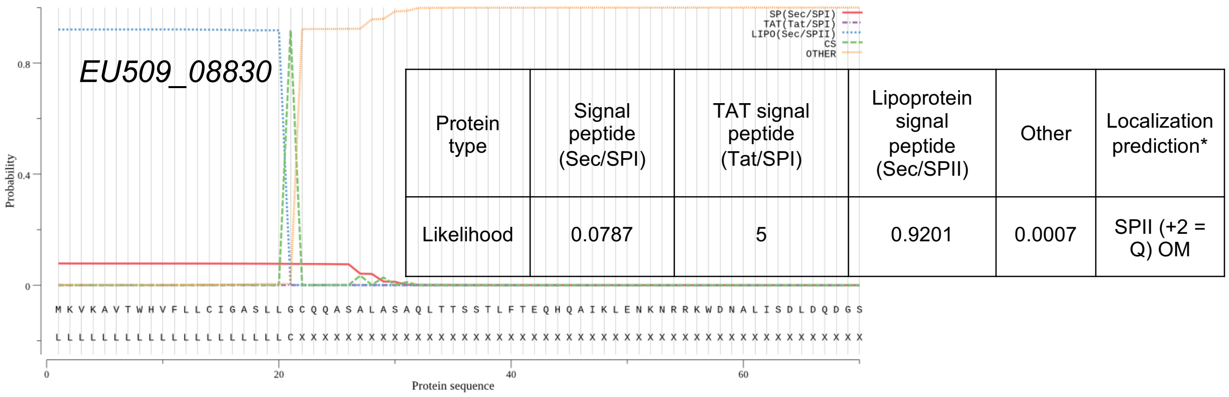

Protein type	Signal peptide (Sec/SPI)	TAT signal peptide (Tat/SPI)	Lipoprotein signal peptide (Sec/SPII)	Other	Localization prediction*
Likelihood	0.0787	5	0.9201	0.0007	SPII (+2 = Q) OM

b

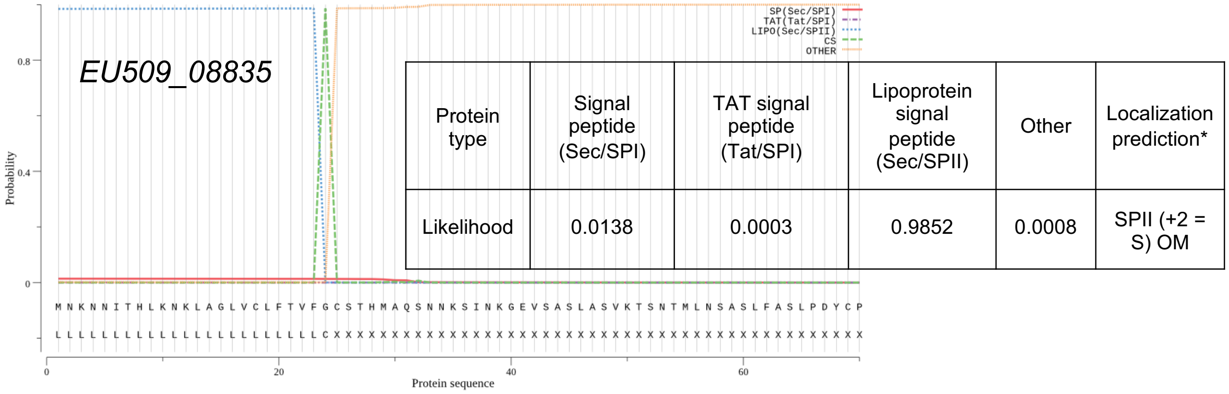

Protein type	Signal peptide (Sec/SPI)	TAT signal peptide (Tat/SPI)	Lipoprotein signal peptide (Sec/SPII)	Other	Localization prediction*
Likelihood	0.0138	0.0003	0.9852	0.0008	SPII (+2 = S) OM

c

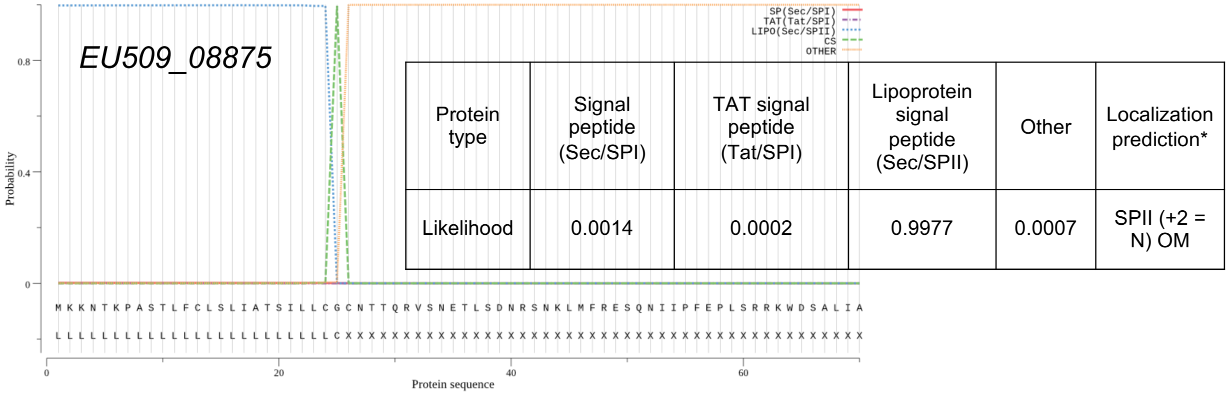

Protein type	Signal peptide (Sec/SPI)	TAT signal peptide (Tat/SPI)	Lipoprotein signal peptide (Sec/SPII)	Other	Localization prediction*
Likelihood	0.0014	0.0002	0.9977	0.0007	SPII (+2 = N) OM

Supplementary Figure 15. Signal peptide prediction in the hypothetical α-1,3-(3,6-anhydro)-D-galactosidase(s) using SignalP [16].

a

Protein	Predicted Fold*	Confidence*	Relevant protein families of same fold
**EU509_8830**	7-bladed β-propeller	100	Lectins, carbohydrate dehydratases (lyase), polysaccharide lyases (families 11, 22, 24, and 25), glycoside hydrolases (families 74 and 145).
**EU509_8835**	6-bladed β-propeller	100	Enzymes of a wide variety of functions including glycoside hydrolases in families 33, 34, 58, 83, and 93.
**EU509_8875**	7-bladed β-propeller	100	Lectins, carbohydrate dehydratase (lyase), polysaccharide lyases (families 11, 22, 24, and 25), glycoside hydrolases (families 74 and 145)

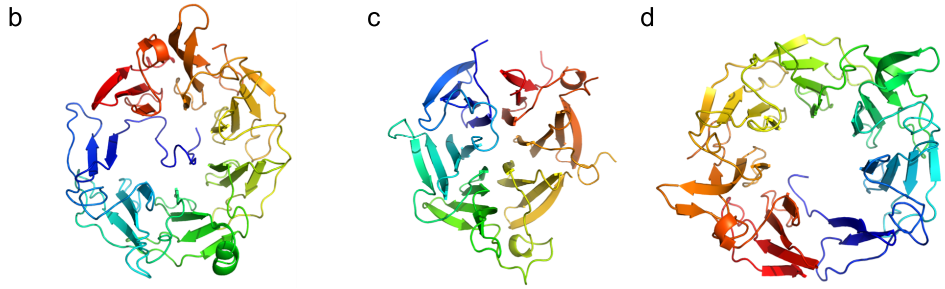

Supplementary Figure 16. Phyre2 fold-prediction analysis of the hypothetical α-1,3-(3,6-anhydro)-D-galactosidase(s). (a) Tabulation of the Phyre2 results. (b-d) Cartoon representations of the folds predicted for EU509_08830, EU509_08835, and EU509_08875, respectively.

Supplementary Table 1: Bacterial isolate and genome details

Strain designation	Species assignment	Total size (bp)	%GC	Best species match by 16S RNA	16S RNA ID (%)	Best species match by genome	OrthoANIu (%)	Number of aligned bases	Presence of carrageenan PUL
PS2*	*Pseudoalteromonas fuliginea* PS2	4,787,931	38.9	*P. fuliginea* KMM216	100	*P. fuliginea* KMM216	98	2,987,151	+
PS42	*Pseudoalteromonas fuliginea* PS42	4,738,574	39.0	*P. fuliginea* KMM216	100	*P. fuliginea* KMM216	98	2,944,109	-
PS47	*Pseudoalteromonas fuliginea* PS47	4,788,025	38.9	*P. fuliginea* KMM216	100	*P. fuliginea* KMM216	98.1	2,981,830	+
FUC4	*Pseudoalteromonas* sp FUC4	4,336,094	39.2	*P. distincta* ATCC 700518	99.8	*P. distincta* ATCC 700518	94.6	2,580,221	+
U2A	*Pseudoalteromonas distincta* U2A	4,476,704	39.2	*P. distincta* ATCC 700518	100	*P. distincta* ATCC 700518	98.2	2,815,175	+

* OrthoANIu of 99.99% over 4,454,130 aligned bases with PS47. Likely independent isolate of the same strain as PS47

Supplementary Table 2: CarPUL gene content and comparison.

Pseudoaltermonas fuliginea PS47				Location prediction		*Pseudoaltermonas fuliginea* PS2		*Pseudoaltermonas* sp. FUC4		*Pseudoaltermonas distincta* U2A		*Pseudoaltermonas carrageenovora 9T*	
Locus Tag	Length (AA)	Annotation/Putative function	Name and Known funcion	LipoP	PSORT	Locus Tag	% AA ID with PS47	PEG	% AA ID with PS47	Locus Tag	% AA ID with PS47	Locus tag	% AA ID with PS47
EU509_08810	794	Glycoside hydrolase, family GH42-like	Exo-β-carrageenase (this study)	SpII (+2 = S) OM	Unknown	EUZ79_08800	100	EU510_02855	93.8	EU511_08810	91.8	PCAR9_p0019	89.8
										(n/a fragment)	73.0 (43 aa)	PCAR9_p0020	71.4 % (43 aa)
EU509_08815	297	Glycoside hydrolase, family 16	Endo-β-carrageenase, κ-carrageenan specific (this study)	SpI	Periplasmic	EUZ79_08805	100	EU510_02860	95.6	EU511_08805	84.0 % (186 aa)	PCAR9_p0021	82.2 (186 aa)
EU509_08820	508	Sulfatase, S1_NC family	Exo-2S-ι-carrageenan sulfatase (this study)	SpI	Periplasmic	EUZ79_08810	100	EU510_02865	97.0	EU511_08800	96.8	PCAR9_p0022	96.8
EU509_08825	481	Endo-4S-iota-carrageenan sulfatase, S1_19 family	Endo-4S-κ/ι-carrageenan sulfatase, S1_19 family (supplementary material reference	SpI	Periplasmic	EUZ79_08815	100	EU510_02870	96.7	EU511_08795	95.8	PCAR9_p0023	95.4
EU509_08830	624	hypothetical protein		SpII (+2 = Q) OM	Unknown	EUZ79_08820	100	EU510_02875	93.4	EU511_08790	93.1	PCAR9_p0024	92.3
EU509_08835	377	conserved hypothetical protein, with a conserved domain		SpII (+2 = S) OM	Unknown	EUZ79_08825	100	EU510_02880	94.0	EU511_08785	93.8	PCAR9_p0025	92.3
EU509_08840	385	hypothetical protein				EUZ79_08830	100						
EU509_08845	137	ABC-type phosphate transport system, periplasmic component				EUZ79_08835	100						
EU509_08850	536	methyl-accepting chemotaxis sensory transducer				EUZ79_08840	100						
EU509_08855	872	hypothetical protein				EUZ79_08845	100						
EU509_08860	1237	TonB-dependent receptor		SpI	Outer membrane	EUZ79_08850	100	EU510_02885	98.8	EU511_08780	98.4	PCAR9_p0026	97.7
EU509_08865	596	Ig-like, group 2		SpII (+2 = G) OM-CM	Extracellular	EUZ79_08855	100	EU510_02890	97.0	EU511_08775	97.0	PCAR9_p0027	89.2
EU509_08870	347	Glycoside hydrolase, family 16	Endo-carrageenase, α/β-carrageenan specific (this study)	SpII (+2 = S) OM	Unknown	EUZ79_08860	100	EU510_02895	90.7	EU511_08770	89.8	PCAR9_p0029	88.0
EU509_08875	643	hypothetical protein		SpII (+2 = N) OM	Unknown	EUZ79_08865	100	EU510_02900	91.0	EU511_08765	91.0	PCAR9_p0030	87.2
EU509_08880	847	TonB-dependent receptor		SpI	Outer membrane	EUZ79_08870	100	EU510_02905	98.1	EU511_08760	97.9	PCAR9_p0031	98.0
EU509_08885	712	TPR domain protein		SpI	Periplasmic	EUZ79_08875	100	EU510_02910	94.1	EU511_08755	94.0	PCAR9_p0033	95.1
EU509_08890	474	Sulfatase, S1_19 family	Exo-2S-κ-carrageenan sulfatase (this study)	SpI	Unknown	EUZ79_08880	100	EU510_02915	97.5	EU511_08750	96.2	PCAR9_p0034	95.1
EU509_08895	388	Galactonate dehydratase		No Sp	Cytoplasmic	EUZ79_08885	100	EU510_02920	97.7	EU511_08745	98.2	PCAR9_p0035	98.5
EU509_08900	292	1,4-lactonase		No Sp	Cytoplasmic	EUZ79_08890	100	EU510_02925	94.2	EU511_08740	92.4	PCAR9_p0036	92.1
EU509_08905	524	MFS transporter/D-xylose proton-symporter XylE		No Sp	Cytoplasmic membrane	EUZ79_08895	100	EU510_02930	98.5	EU511_08735	98.5	PCAR9_p0037	97.7
EU509_08910	260	SDR family oxidoreductase		No Sp	Cytoplasmic	EUZ79_08900	100	EU510_02935	98.5	EU511_08730	98.8	PCAR9_p0038	98.1
EU509_08915	328	conserved hypothetical protein		No Sp	Cytoplasmic	EUZ79_08905	100	EU510_02940	95.7	EU511_08725	85.6	PCAR9_p0039	85.6
EU509_08920	476	3,6-anhydro-D-galactose dehydrogenase (DauA)	3,6-anhydro-D-galactose dehydrogenase (this study)	No Sp	Cytoplasmic	EUZ79_08910	100	EU510_02945	98.3	EU511_08720	93.3	PCAR9_p0040	92.0
EU509_08925	388	3,6-anhydro-D-galactonate cycloisomerase (DauB)		No Sp	Cytoplasmic	EUZ79_08915	100	EU510_02950	99.7	EU511_08715	98.2	PCAR9_p0041	97.4
EU509_08930	336	2-keto-3-deoxy-D-galactonate kinase (DauC)		No Sp	Cytoplasmic	EUZ79_08920	100	EU510_02955	96.4	EU511_08710	77.6	PCAR9_p0042	78.2
EU509_08935	207	2-keto-3-deoxy-D-galactonate aldolase (DauD)		No Sp	Cytoplasmic	EUZ79_08925	100	EU510_02960	98.1	EU511_08705	91.3	PCAR9_p0043	92.7
EU509_08940	231	D-Galactonate repressor DgoR		No Sp	Cytoplasmic	EUZ79_08930	100	EU510_02965	97.0	EU511_08700	94.8	PCAR9_p0044	94.4
EU509_08945	246	D-Galactonate repressor DgoR		No Sp	Cytoplasmic	EUZ79_08935	100	EU510_02970	99.6	EU511_08695	99.6	PCAR9_p0045	98.8
EU509_08950	947	TonB-dependent receptor		SpI	Outer membrane	EUZ79_08940	100	EU510_02975	94.9	EU511_08690	93.5	PCAR9_p0046	93.3
EU509_08955	404	Transcriptional regulator, AraC family		No Sp	Cytoplasmic	EUZ79_08945	100			EU511_08685	77.6	PCAR9_p0047	77.2
EU509_08960	398	Glycoside hydrolase, family 16	Endo-β-carrageenase, κ-carrageenan specific, CgkA (this study and supplementary material references 3 and 4)	SpI	Periplasmic	EUZ79_08950	100			EU511_08680	91.7	PCAR9_p0048	91.7
EU509_08965	323	hypothetical protein		No Sp	Cytoplasmic	EUZ79_08955	100			EU511_08675	71.5	PCAR9_p0049	71.7
EU509_08970	256	2-keto-4-pentenoate hydratase		No Sp	Cytoplasmic	EUZ79_08960	100	EU510_02980	77.3	EU511_08670	69.4	PCAR9_p0050	68.7

- *Glycoside hydrolase*
- *Sulfatase*
- *Candidate α-1,3-(3,6-anhydro)-D-galactosidase*
- *TonB-dependent receptor (TBDR)*
- *Other function or hypothetical protein*
- *Galactose/Galactonate metabolism*
- *Major facilitator superfamily sugar transporter (MFS)*
- *Transcriptional regulator*

Supplementary Table 3: Oligonucleotide primer sequences used for gene amplification

S1_NC_fwd	5'-CATATGGCTAGCATCAAAAAGCCAAATGTGC -3'
S1_NC_rev	5'-GTGTGTCTCGAGCTATAAAAAAGCTTTCATGTGGTG-3'
S1_NC_fwd_C84S	5'-CAGGCTTATCCTCACCTTCGCGC-3'
S1_NC_rev_C84S	5'-AGGTGAGGATAAGCCTGTAGATG-3'
S1_NC_fwd_C84A	5'-CAGGCTTAGCCTCACCTTCGCG-3'
S1_NC_rev_C84A	5'-AGGTGAGGCTAAGCCTGTAGATGTC-3'
S1_19B_fwd	5'-CATATGGCTAGCAAACCTAATATTGTTTTAATTTTTGC-3'
S1_19B_rev	5'-GTGTGTCTCGAGTTAAGGTTGAGTTGCTGGTAAAC-3'
S1_19B_fwd_C77S	5'-GTGATTCTACTTCTGGTCCTTCACGCGCAG-3'
S1_19B_rev_C77S	5'-GTGAAGGACCAGAAGTAGAATCACTCACGTAAC-3'
GHnew_fwd	5'-AAGGAGATATACCATGTCGCAGCAAGAAAGCG-3'
GHnew_rev	5'-GTGGTGGTGGTGGTGTTGAACAGTAACTTCAACAAGC-3'
Vector_primer1	5'-GCTTTCTTGCTGCGACATGGTATATCTCCTTCTTAAAGTTAAAC-3'
Vector_primer2	5'-GAAGTTACTGTTCAACACCACCACCACCACC-3'
BovGHnew_fwd	5'-CATATGGCTAGCGGTATCAACTACCTGTA-3'
BovGHnew_rev	5'-GTGTGTCTCGAGTCATTCCAGGCTCACTT-3'
DauA_fwd	5'-AGCCATATGGCTAGCCTTTCTAAACAACTGTATATCGGTGGAA-3'
DauA_rev	5'-GTGGTGGTGCTCGAGTTAAACCTTTAACGCGCGAGAAAC-3'
H6 F MGH16A MSC amp	5'-GTTAAAGCAACACCACCACCACCACCACTG-3'
MalE R MGH16A MSC amp	5'-TCGGTTGGCTCTGGAAGTACAGGTTCTCGG-3'
MGH16A GH16A IF F	5'-TCCAGAGCCAACCGATGCCACATATTGC-3'
MGH16A GH16A IF R	5'-TGGTGTTGCTTTAACCATGTTCTCACGTAATCAAC-3'
H6 F MGH16B MSC amp	5'-GTCAGTTAAGCACCACCACCACCACC-3'
MalE R MGH16B MSC amp	5'-GTATTACTGCTCTGGAAGTACAGGTTCTCG-3'
MGH16B GH16B IF F	5'-CCAGAGCAGTAATACGCAAAAGAGCCTGCGTGTT-3'
MGH16B GH16B IF R	5'-TGGTGCTTAACTGACTGTATTGGTTTTTGCTGCCA-3'
H6 F MGH16C MSC amp	5'-AACGGTGAATCACCACCACCACCACCACTG-3'
MalE R MGH16C MSC amp	5'-GTATATCAGCGCTCTGGAAGTACAGGTTCTCGGTG-3'
MGH16C GH16C IF F	5'-AGAGCGCTGATATACAACCACCTATTGCTAAACC-3'
MGH16C GH16C IF R	5'-TGGTGATTCACCGTTATGATTAATTTATCTGTTTTACCTT-3'

Supplementary Table 4: Crystallization condition for all X-ray crystal structures

Protein	[Protein] (mg/ml)	Precipitant (w/v)	Buffer	Salt	Additive
S1_19B Native	20	25% PEG 3350	0.1 M sodium acetate (NaOAc) trihydrate (pH 4.5)	-	-
S1_19B C77S ι-NC4 and κ-NC2 complexes	20	20 – 21% PEG 3350	-	0.02 – 0.05 M $ZnCl_2$	0.1 M gly-gly 7.5 % glycerol
S1_NC wild type	24	25% PEG 5K MME	0.1 M Bis Tris (pH 6.5)	-	0.1 M 2-Methyl-2,4-pentanediol (MPD)
S1_NC C84S ι-NC2 and κ-NC2 complexes	20	20 – 21% PEG 4000	0.1 M HEPES (pH 7.5)	0.05 – 0.075 M NaOAc	0.1 M arginine 10 % glycerol
S1_NC C84A SO_4^{2-} complex	11	12.4% PEG 8000	0.08 M Na cacodylate (pH 6.4)	0.075 M Ca acetate	0.1 M $LiCl_2$ 20 % Glycerol
BovGH42L Native	20	30% PEG 400	0.1 M Tris (pH 8.5)	0.2 M $MgCl_2$	-
BovGH42L Iodide derivative	20	25% PEG 4000	0.1 M tri-sodium citrate (pH 5.6)	0.2 M ammonium sulfate	-
DauA	22	25% PEG 3350	-	0.3 M ammonium fluoride	-
DauA with NADP+	22	20% PEG 3350	0.2 M sodium acetate trihydrate and 0.1 M Bis-Tris pH 6.0	-	-

Supplementary Table 5: X-ray data collection and structure statistics

	S1_19B	S1_19B C77S	S1_19B C77S
	Native	κ-NC2 complex	ι-NC4 complex
Data Collection			
Beamline	CLS 08ID-1	CLS 08ID-1	In-house
Wavelength	0.9794	0.9794	1.5418
Space Group	P1	$P3_1$	$P2_12_12_1$
Cell Dimensions			
a, b, c (Å)	48.51, 88.58, 106.6	93.05, 93.05, 300.21	65.26, 94.00, 170.55
Resolution (Å)	47.60-2.50 (2.57-2.50)	48.15-1.95 (1.98-1.95)	30.00-2.00 (2.03-2.00)
R_{merge}	0.108 (0.334)	0.279 (1.178)	0.050 (0.087)
R_{pim}	0.103 (0.317)	0.299 (1.278)	0.023 (0.062)
CC1/2	0.989 (0.861)	0.995 (0.824)	0.997 (0.983)
$\langle I/\sigma I \rangle$	8.4 (3.3)	12.6 (3.2)	25.6 (9.2)
Completeness (%)	97.9 (96.4)	99.7 (99.1)	99.6 (98.8)
Redundancy	3.8 (3.3)	11.6 (11.6)	3.7 (2.4)
No. of Reflections	220,121	2,443,240	261,729
No. Unique	58,432	211,230	70,964
Refinement			
Resolution (Å)	2.50	1.95	2.00
R_{work}/R_{free}	0.24/0.27	0.19/0.21	0.20/0.23
No. of Atoms			
Protein	3497 (A), 3497 (B),<br>3505 (C), 3498 (D)	3537 (A), 3526 (B),<br>3540 (C), 3505 (D),<br>3545 (E), 3522 (F)	3544 (A), 3545 (B)
Ligand	4 (Ca)	6 (Ca),<br>26 (κ-NC2 A), 26 (κ-NC2 B),<br>26 (κ-NC2 C), 26 (κ-NC2 D),<br>26 (κ-NC2 E), 26 (κ-NC2 F),<br>6 (Cl), 6 (Zn),<br>20 (EDO)	2 (Ca), 2(Cl),<br>59 (ι-NC4 A),<br>59 (ι-NC4 B)
Water	104	886	344
B-factors			
Protein	34.1 (A), 33.0 (B),<br>33.6 (C), 35.8 (D)	20.5 (A), 22.9 (B),<br>19.8 (C), 23.1 (D),<br>21.8 (E), 18.4 (F)	23.6 (A), 27.1 (B)
Ligand	37.2 (Ca)	28.6 (Ca),<br>18.0 (κ-NC2 A),<br>19.1 (κ-NC2 B),<br>17.0 (κ-NC2 C),<br>22.2 (κ-NC2 D),<br>18.8 (κ-NC2 E),<br>15.8 (κ-NC2 F),<br>19.2 (Cl), 22.2 (Zn),<br>26.7 (EDO)	17.6 (Ca), 28.9 (Cl),<br>31.4 (ι-NC4 A),<br>30.9 (ι-NC4 B)
Water	19.3	20.0	25.3
r.m.s.d.			
Bond Lengths (Å)	0.007	0.008	0.007
Bond Angles (°)	1.426	1.430	1.548
Ramachandran (%)			
Preferred	95.3	96.8	96.3
Allowed	4.7	3.2	3.7
Disallowed	0	0	0

	S1_NC<br>Native	S1_NC C84S<br>ι-NC4 complex	S1_NC C84S<br>κ-NC2 complex
Data Collection			
Beamline	CLS 08ID-1	In-house	SSRL B11-1
Wavelength	0.9787	1.5418	0.9795
Space Group	$P2_12_12_1$	$P2_12_12_1$	$P2_12_12_1$
Cell Dimensions			
a, b, c (Å)	57.47, 102.09, 170.78	57.72, 102.44, 171.21	57.69, 101.79, 170.54
Resolution (Å)	49.72-1.45 (1.47-1.45)	50.00-2.25 (2.29-2.25)	39.33-1.66 (1.75-1.66)
R_{merge}	0.062 (0.253)	0.096 (0.292)	0.076 (0.603)
R_{pim}	0.037 (0.149)	0.042 (0.154)	0.028 (0.244)
CC1/2	0.999 (0.960)	0.994 (0.920)	0.999 (0.814)
<I/σI>	16.6 (6.0)	14.6 (3.5)	20.5 (3.1)
Completeness (%)	100.0 (100.0)	95.3 (94.9)	99.9 (99.2)
Redundancy	7.3 (7.5)	4.0 (3.5)	8.3 (6.9)
No. of Reflections	1,304,490	189,172	989,254
No. Unique	178,310	46,858	119,231
Refinement			
Resolution (Å)	1.45	2.25	1.66
R_{work}/R_{free}	0.14/0.16	0.20/0.25	0.18/0.20
No. of Atoms			
Protein	3788 (A), 3797 (B)	3762 (A), 3751 (B)	3761 (A), 3779 (B)
Ligand	2 (Ca), 6 (Cl),<br>44 (EDO)	2 (Ca), 30 (ι-NC4 A),<br>30 (ι-NC4 B)	2 (Ca),<br>26 (κ-NC2 A),<br>26 (κ-NC2 B),<br>8 (EDO), 15 (EPE)
Water	803	312	675
B-factors			
Protein	10.3 (A), 9.1 (B)	33.2 (A), 33.4 (B)	18.7 (A), 16.7 (B)
Ligand	5.9 (Ca), 13.5 (Cl),<br>19.0 (EDO)	37.4 (Ca),<br>50.6 (ι-NC4 A),<br>59.2 (ι-NC4 B)	12.4 (Ca),<br>20.4 (κ-NC2 A),<br>17.5 (κ-NC2 B),<br>25.3 (EDO),<br>31.7 (EPE)
Water	17.4	29.2	22.1
r.m.s.d.			
Bond Lengths (Å)	0.014	0.011	0.011
Bond Angles (°)	1.827	1.680	1.649
Ramachandran (%)			
Preferred	98.3	98.0	98.0
Allowed	1.7	2.0	2.0
Disallowed	0	0	0

	S1_NC C84A SO_4^- complex	BovGH42L Native	BovGH42L Iodide derivative
Data Collection			
Beamline	CLS 08ID-1	SSRL B11-1	SSRL BL11-1
Wavelength	0.9794	1.1271	1.3776
Space Group	C121	$P2_12_12_1$	$P2_12_12_1$
Cell Dimensions			
a, b, c (Å)	124.83, 110.68, 175.90	60.25, 109.68, 126.1	60.54, 110.16, 125.61
Resolution (Å)	68.78-1.75 (1.84-1.75)	54.66-2.00 (2.05-2.00)	39.14-2.38 (2.47-2.38)
R_{merge}	0.099 (0.202)	0.128 (0.682)	0.238 (1.199)
R_{pim}	0.055 (0.112)	0.077 (0.438)	0.066 (0.382)
CC1/2	0.990 (0.948)	0.996 (0.729)	0.994 (0.689)
$\langle I/\sigma I\rangle$	9.8 (5.7)	11.2 (2.4)	10.2 (2.1)
Completeness (%)	87.0 (84.0)	99.9 (99.5)	99.6 (96.4)
Redundancy	4.0 (4.2)	7.0 (6.3)	14.0 (10.2)
No. of Reflections	814,317	401,461	479,400
No. Unique	201,748	57,229	34,182
Refinement			
Resolution (Å)	1.75	2.00	
R_{work}/R_{free}	0.14/0.16	0.17/0.20	
No. of Atoms			
Protein	3768 (A), 3784 (B), 3769 (C), 3770 (D)	5814	
Ligand	14 (Ca), 26 (κ-C3 A), 26 (κ-C3 B), 26 (κ-C3 C), 26 (κ-C3 D), 54 (GOL), 40 (EDO), 20 (SO_4)	20 (EDO)	
Water	1843	418	
B-factors			
Protein	15.3 (A), 13.1 (B), 14.2 (C), 13.8 (D)	21.5	
Ligand	12.3 (Ca), 21.9 (κ-C3 A), 22.3 (κ-C3 B), 23.9 (κ-C3 C), 20.8 (κ-C3 D), 15.9 (GOL), 23.3 (EDO), 9.3 (SO_4)	26.7 (EDO)	
Water	23.2	24.4	
r.m.s.d.			
Bond Lengths (Å)	0.012	0.010	
Bond Angles (°)	1.635	1.614	
Ramachandran (%)			
Preferred	97.7	97.0	
Allowed	2.1	3.0	
Disallowed	0.2	0	

	DauA<br>Native	DauA<br>NADP$^+$ complex	
Data Collection			
Beamline	CLS 08ID-1	CLS 08ID-1	
Wavelength	0.9795	0.9795	
Space Group	$P2_1$	$P2_1$	
Cell Dimensions			
a, b, c (Å)	82.9, 137.8, 108.9<br>β=109.9	87.7, 232.8, 88.3,<br>β=119.2	
Resolution (Å)	75.66-2.00 (2.03-2.00)	39.85-2.15 (2.18-2.15)	
R_{merge}	0.146 (0.656)	0.136 (0.579)	
R_{pim}	0.075 (0.346)	0.072 (0.328)	
CC1/2	0.984 (0.818)	0.989 (0.671)	
$<I/\sigma I>$	5.9 (2.3)	9.0 (2.1)	
Completeness (%)	99.4 (98.9)	99.2 (98.1)	
Redundancy	5.3 (5.3)	5.4 (5.0)	
No. of Reflections	820,225	895,796	
No. Unique	153,728	167,353	
Refinement			
Resolution (Å)	2.00	2.15	
R_{work}/R_{free}	0.22/0.26	0.21/0.26	
No. of Atoms			
Protein	3561 (A), 3569(B),<br>3550 (C), 3576 (D),<br>35 (E), 43 (F), 40 (H)	3556 (A), 3542 (B),<br>3544 (C), 3535 (D),<br>3543 (E), 3531 (F)	
Ligand	80 (EDO)	288 (NAP), 56 (EDO),<br>2 (MG)	
Water	771	564	
B-factors			
Protein	34.7 (A), 29.6 (B),<br>29.4 (C), 28.2 (D),<br>50.0 (E), 45.1 (F), 43.1 (H)	35.3 (A), 36.2 (B),<br>35.1 (C), 35.8 (D),<br>37.2 (E), 37.7 (F)	
Ligand	39.5 (EDO)	34.2 (NAP), 42.3 (EDO),<br>43.7 (MG)	
Water	33.2	35.0	
r.m.s.d.			
Bond Lengths (Å)	0.003	0.002	
Bond Angles (°)	0.513	0.543	
Ramachandran (%)			
Preferred	96.5	96.7	
Allowed	3.5	3.3	
Disallowed	0.0	0.0	

Supplementary Table 6. List of major ions observed in ι-NC4 and its digestion products

Major molecular and fragment ions*	Accurate mass observed (m/z)	Composition	Error (ppm)
$[(Tet\text{-}3S)]^{3-}$	289.01694	C24H35O28S3	1.704
$[(Tet\text{-}4S)H]^{3-}$	315.66921	C24H35O31S4	1.556
$[(Tet\text{-}2S)]^{2-}$	394.05038	C24H36O25S2	1.213
$[(Tet\text{-}3S)H]^{2-}$	434.02904	C24H36O28S3	1.683
$[(Tet\text{-}3S)NH_4]^{2-}$	442.54236	C24H39O28NS3	1.754
$[(Tet\text{-}3S)Na]^{2-}$	445.02003	C24H35O28NaS3	1.681
$[(Tet\text{-}3S)K]^{2-}$	453.00701	C24H35O28KS3	1.677
$[(Tet\text{-}4S)2H]^{2-}$	474.00750	C24H36O31S4	1.653
$[(Tet\text{-}4S)H+NH_4]^{2-}$	482.52080	C24H39O31NS4	1.676
$[(Tet\text{-}4S)\ H+K]^{2-}$	492.98560	C24H35O31KS4	1.912
$[(Tet\text{-}4S)2K]^{2-}$	511.96345	C24H34O31K2S4	1.663
$[(Tet\text{-}1S)]^{-}$	709.15111	C24H37O22S	1.190
$[(Tet\text{-}2S)H]^{-}$	789.10924	C24H37O25S2	2.736
$[(Tet\text{-}2S)NH_4]^{-}$	806.13562	C24H40O25NS2	2.469
$[(Tet\text{-}2S)Na]^{-}$	811.09151	C24H36O25NaS2	3.063
$[(Tet\text{-}2S)K]^{-}$	827.06545	C24H36O25KS2	3.007
$[(Tet\text{-}3S)2H]^{-}$	869.06625	C24H37O28S3	2.709
$[(Tet\text{-}3S)H+NH_4]^{-}$	886.09323	C24H40O28NS3	3.144
$[(Tet\text{-}3S)2NH_4]^{-}$	903.11973	C24H43O28N2S3	3.030
$[(Tet\text{-}3S)H+K]^{-}$	907.02275	C24H36O28KS3	3.277
$[(Tet\text{-}3S)Na+K]^{-}$	929.00449	C24H35O28KNaS3	2.980
$[(Tet\text{-}3S)2K]^{-}$	944.97849	C24H35O28K2S3	2.996
$[(Tet\text{-}4S)2H+NH_4]^{-}$	966.05054	C24H40O31NS4	3.396

$[(Tet\text{-}4S)2H+K]^-$	986.98011	C24H36O31KS4	3.565
$[(Tet\text{-}4S)3NH_4]^-$	1000.10446	C24H46O31N3S4	4.102
$[(Tet\text{-}4S)H+2K]^-$	1024.93589	C24H35O31K2S4	3.333
$[(Tet\text{-}4S)Na+2K]^-$	1046.91806	C24H34O31K2NaS4	3.478
$[(Tet\text{-}4S)3K]^-$	1062.89213	C24H34O31K3S4	3.551

*Tet refers to the backbone tetrasaccharide of 3,6-anhydro-D-galactose (DA) and D-galactose (G) comprising DA-G-DA-G. This is followed by the number of sulfate groups present on the tetrasaccharide backbone.

Supplementary Notes

1. Genomic analysis. Though PS2 and PS47 were independently isolated and sequenced instances of the same strain, their CarPULs were found on identically sized contigs of 135169 bp. This suggests that the CarPUL is present on a plasmid, similar to the 143 kbp plasmid in *Pseudoalteromonas carrageenovora* 9^T that bears the CarPUL [1]. Likewise, the U2A CarPUL is on a contig of 111950 bp and, like in *P. carrageenovora* 9^T, the CarPUL is immediately preceded by genes encoding predicted ParA and RepA-like proteins, suggesting the U2A CarPUL is also on a plasmid. In contrast, however, in FUC4 the CarPUL is present on a contig of >1.4 Mbp, mostly likely indicating its presence on a chromosome.

2. Primary structure analysis and modeling of GH16 enzymes. The only GH16 conserved between *P. carrageenovora* 9^T and all of our *Pseudoalteromonas* strains is GH16B, which is predicted to be a lipoprotein localized to the outer membrane. GH16B shows only ~25% amino acid sequence identity with both GH16A and GH16C, but ~78% amino acid sequence identity with a *Paraglaciecola hydrolytica* $S66^T$ GH16 enzyme that is classified as a β-carrageenan specific *endo*-hydrolase [2]. The gene encoding GH16A, which is predicted to be a periplasmic protein, is also present in the *Pseudoalteromonas* strains, though the gene is fragmented in *P. carrageenovora* 9^T and U2A. With 92% amino acid sequence identity over their entire lengths, GH16C is the orthologue of the well characterized κ-carrageenanase CgkA (PCAR9_p0048), which is responsible for the secreted κ-carrageenanase activity in *P. carrageenovora* 9^T [1,3–6] and therefore possibly also in PS47. GH16A and GH16C have 76% amino acid sequence identity; however, GH16A lacks the ~100 amino acid C-terminal domain present in GH16C and CgkA (Supplementary Figure 4a). Modeling the structures of GH16A and GH16C using CgkA as a template indicate 100% conservation of the active sites, pointing to conserved specificity amongst these enzymes (Supplementary Figure 4b).

3. Structural analysis of S1_19B. The structure of the wild type S1_19B enzyme at 2.50 Å resolution was solved using S1_19A from PS47 (PDB ID code: 6BIA)[7] as the search model. The final refined model contained four molecules in the asymmetric unit arranged

as a tetramer (Supplementary Figure 7b). PISA analysis[8] of the S1_19B structure predicts the tetramer to be composed of two dimers, with buried surface areas of ~1460 Å^2 between the chains participating in the dimer interface (chains A and B, or C and D), and only ~362 Å^2 between chains from the different dimers (i.e. between chains A and C). The molecular interactions of the dimer interfaces show an extensive and direct hydrogen bond network (Supplementary Figure 7b inset). To assess the oligomeric state of S1_19B in solution, the protein was passed through a calibrated size exclusion chromatography column. The elution volume for S1_19B corresponds to an experimental molecular weight of 91.3 kDa (Supplementary Figure 7c). This is between the expected molecular weight of the S1_19B monomer (55.828 kDa) and dimer (112 kDa), but is closer to that of a dimer. The PISA analysis and the elution volume results support the dimer as being the biological unit for S1_19B. In contrast, the other S1_19 family member in the CarPUL, S1_19A, adopts a stable trimeric structure [7].

4. Structural analysis of S1_NC. The final refined model of native S1_NC contained two molecules in the asymmetric unit arranged as a homodimer with C2 symmetry (Supplementary Figure 8a). PISA analysis of this structure predicts the dimer to be a stable oligomeric state with ~1500 Å of buried surface area at the dimer interface. This interface is composed of an extensive and symmetrical hydrogen bond network (Supplementary Figure 8a inset). The formation of a physiologically relevant dimer was further supported by size exclusion chromatography analysis of S1_NC, which gave an elution volume corresponding to an experimental molecular weight of 102.3 kDa (Supplementary Figure 8b). With an expected molecular weight of 56.613 kDa for a monomer and 113.2 kDa for a dimer, this experimental value is most consistent with that of a dimer rather than a monomer.

The X-ray crystal structure of native S1_NC showed unusual electron density at the proto-catalytic C84 residue (Supplementary Figure 9a). Efforts at modelling this density revealed that it was best modeled as a cysteinesulfinic acid residue, which likely represents the oxidized form of the cysteinesulfenic acid intermediate proposed in the normal maturation of the catalytic FGly residue (Supplementary Figure 9b-g)[9]. Despite

extensive efforts, we were unable to circumvent generation of this inappropriately matured form of the protein. Therefore, to provide insight into the specificity of this enzyme we generated C84A and C84S mutants, which prevented post-translational modification of the catalytic site, and attempted to determine structures of these mutants in complex with neocarrageenan oligosaccharides and fragments thereof. These initial efforts led to separate structures of S1_NC C84A and S1_NC C84S in complex with a sulfate and κ-NC2, respectively (Supplementary Figures 10a and 10b). Superimposition of these structures revealed a composite mimicking a product complex of ι-NC2 hydrolysis whereby 0 and +1 subsites were occupied by the DA and G4S residues, respectively, of the κ-NC2 "product," while the S-subsite was occupied by a free sulfate found in proximity to the O2 of the DA residue (Supplementary Figure 10c). Informed by this, we subsequently determined the structure of S1_NC C84S in complex with ι-NC4 (Supplementary Figure 10d, see also main text).

5. Characterization of the 3,6-anhydro-D-galactose dehydrogenase, DauA. Recombinant DauA from PS47 had no significant activity on DA when using NAD^+ as a co-factor. However, it displayed activity on DA when using $NADP^+$ as a co-factor and had optimum activity between pH 7.5 and 8.0 (Supplementary Figure 12). At pH 7.5 the K_m and k_{cat} values were 442.4 (±42.3) μM and 30.6 (±1.4) s^{-1}, respectively, for DA. The corresponding values for $NADP^+$ were 147.1 (±13.5) μM and 21.5 (±0.6) s^{-1}.

DauA was crystallized in two distinct crystal forms, each in the space group $P2_1$. Native DauA, whose structure was determined by molecular replacement, crystallized with four molecules in the asymmetric, while DauA in complex with $NADP^+$ crystallized with six molecules in the asymmetric unit. The common quaternary structure of the two crystal forms, which is predicted by PISA analysis to be stable, is a dimer.

In the $NADP^+$ complex, the 2 prime-sulfate group of the adenine group is accommodated in a pocket where two direct and two water mediated hydrogen bonds are made between the sulfate and the protein (Supplementary Figure 13b). The preference of DauA for $NADP^+$ over NAD^+ distinguishes the *Pseudoalteromonas* enzyme from ZgDauA, which

prefers NAD^+ [10]. Notably, S174 of DauA, which provides the end-wall of the sulfate binding pocket, is substituted by a glutamic acid in ZgDauA. Additionally, D204, which in DauA provides a hydrogen bond with the sulfate, is substituted by a valine in ZgDauA (Supplementary Figure 13c). These changes may provide steric hindrance with the 2 prime-sulfate of $NADP^+$ and a loss of H-bonding potential in ZgDauA, possibly explaining the difference in co-factor preference between the two enzymes.

6. Activity of S1_19A on ι-NC4. Our previous characterization of S1_19A indicated that this enzyme is primarily an *endo*-acting 4S-sulfatase that prefers ι-carrageenan[7]. However, the enzyme displayed the ability to process ι-NC4, but not ι-NC2, and had very low activity on κ-carrageenan oligosaccharides with a degree of polymerization of four or more. X-ray crystallographic and NMR analyses of the interaction of S1_19 with substrate indicated its specificity for G4S residues. Furthermore, the poise of ι-NC4 in the crystal structure of the S1_19A ι-NC4 complex showed recognition of the non-reducing end ι-neocarrabiose motif in all three of the protein monomers in the asymmetric unit. Given the ability to process an ι-carrageenan oligosaccharide and the role this could potentially play in the *Pseudoalteromonas* pathway of carrageenan processing we further probed ι-NC4 processing by mass spectrometry. Profiling of oligosaccharide species in ι-NC4 and its digestion products treated with sulfatase for 1 or 2 hrs was carried out using a LC-HRMS/DAD/ELSD method, with a graphitized carbon column as reported[11]. The starting ι-NC4 substrate showed predominantly masses consistent with a tetrasaccharide having four sulfate groups, as expected for ι-NC4 (Supplementary Figure 14a and 14b, and Supplementary Table 7). Minor amounts of di- and tri-sulfated species were detected and are likely due to ionization induced neutral loss of sulfate groups.

After one and two hours of incubating ι-NC4 with S1_19A a new product was formed with a different retention time; this was the same for both samples so only the 2 hour sample was used for further analysis and comparison (Supplementary Figure 14c). The product showed predominantly masses consistent with a tetrasaccharide having three sulfate groups, indicating the enzymatic removal of a single sulfate group (Supplementary Figure

14d and Supplementary Table 6). Minor amounts of di-sulfated species were detected, which are also likely due to ionization induced neutral loss of sulfate groups. Thus, combining the mass spectrometry and structural data, our interpretation is that S1_19A is capable of removing the 4-sulfate from the G4S residue adjacent to the non-reducing end, but is unlikely to desulfate all G4S residues in an oligosaccharide.

7. Analysis of EU509_8830, EU509_8835, and EU509_8875 – hypothetical α-1,3-(3,6-anhydro)-D-galactosidases. As an initial step towards identifying the α-1,3-(3,6-anhydro)-D-galactosidases we focused on putative proteins that a) did not have an experimentally identified function, b) did not have a confident bioinformatic prediction, and c) was conserved amongst all of the strains. This narrowed the possibilities to EU509_08830, EU509_08835, EU509_08865, EU509_08875, EU509_08885, and EU509_08915. Fold prediction for EU509_08915 using Phyre2 [12] returns a mutarotase-like fold with 100% confidence, which interpreted in light of genomic context, where the gene residues in the monosaccharide processing cluster (Supplementary Table 2), and prediction of cytoplasmic localization (Supplementary Table 2) is most consistent with the protein acting as a galactose or 3,6-anhydro-D-galactose mutarotase. EU509_08865 is predicted to be a secreted lipoprotein. Its fold, however, suggests the presence of non-catalytic carbohydrate-binding modules indicating that this protein is may be a SusE-like protein that mediates adherence to polysaccharides. Fold recognition analysis of EU509_08885 indicates it contains a domain with a cytochrome C-like fold followed by a tetratricopeptide repeat (TPR) domain, both with >99.9% confidence; neither fold is consistent with activity on glycosidic bonds. Rather, the similarity of this fold to a recently identified galactose demethylase suggests this may be the function of EU509_08885 [13]. The likelihood of these three proteins having functions other than glycosidic bond cleavage then leaves EU509_08830, EU509_08835, and EU509_08875, which are all predicted to be secreted lipoproteins (Supplementary Figure 15). EU509_08830 and EU509_08875, which share 38% amino acid sequence identity, are predicted with 100% confidence by Phyre2 to have a 7-bladed β-propeller fold (Supplementary Figure 16). This fold is associated with a variety of different functions, but, notably, is found in carbohydrate specific lectins, polysaccharide lyases, and glycoside hydrolases, latter two of which are classes of

enzymes that cleave glycosidic bonds. Similarly, the 6-bladed β-propeller fold predicted to be adopted by EU509_8835 is also associated with a variety of functions, including glycoside hydrolysis (Supplementary Figure 16). On this basis, we favor EU509_08830, EU509_08835, and EU509_08875 as the most likely candidates for the α-1,3-(3,6-anhydro)-D-galactosidase(s) in the CarPUL.

8. Comparison of agarose and carrageenan degradation pathways. There are two general models proposed for saccharification of agarose, which is an algal galactan that is related to carrageenan but is typically non-sulfated (or less sulfated) and has 3,6-anhydro-L-galactose in place of DA. Both models rely on initiation of depolymerization by the action of *endo*-acting β-agarases to generate a pool of neoagaroligosaccharides. One model, which we refer to as the "*exo* model," then relies on the sequential action of an *exo*-α-1,3-L-neoagarooligosaccharide hydrolase (GH117) and an *exo*-β-D-galactosidase (GH2) to reduce the oligosaccharides to monosaccharides [14]. The other model uses a neoagarobiose releasing β-D-agarase (GH50) to reduce neoagarooligosaccharides to neogarobiose, which is then followed by hydrolysis to monosaccharides by a GH117 [15]. The *Pseudoalteromonas* CarPUL does not contain any genes encoding a candidate *exo*-β-D-galactosidase, such as the GH2 enzymes of *Z. galactanivorans* and *Paraglaciecola hydrolytica* S66[T] that have *exo*-β-galactosidase activity on the β-1,4-linkages in carrageenan [2,10], but instead employs a β-NC2 releasing β-carrageenanase. Therefore, the model of ι/κ-carrageenan metabolism by our pseudoalteromonad isolates most closely parallels the latter model of agarose metabolism. In contrast to the pseudoalteromonad model, the pathway proposed for ι/κ-carrageenan metabolism by *Z. galactanivorans* utilizes a parallel of the "*exo* model" [10].

In both models of carrageenan depolymerization a key step is the hydrolysis of the non-reducing terminal α-1,3-(3,6-anhydro)-D-galactose residue. While the α-1,3-(3,6-anhydro)-D-galactosidase has not yet been identified in CarPUL-containing *Pseudoalteromonas* species, their growth phenotype on κ- and ι-carrageenan indicates that this enzyme activity must be present. The failure to identify candidate enzymes

belonging to GH families 127 and 129 indicates that the pseudoalteromonad solution to hydrolyzing the α-glycosidic linkage in carrageenan is different than that of *Z. galactanivorans,* where this activity is clearly attributed to GH127 and GH129 enzymes*,* thus highlighting another difference between the two pathways.

With respect to the sulfatases employed, which are a notable feature of carrageenan metabolism, the two characterized pathways use different complements of enzymes. The *Pseudoalteromonas endo*-acting sulfatase S1_19A and *Z. galactanivorans* ZGAL_3145 (family S1_19) display very similar biochemical properties: activity most consistent with *endo*-G4S ι-carrageenan sulfatase specificity and the capability of producing α-carrageenan on the non-reducing end of an ι-carrageenan oligosaccharide*.* The κ-carrageenan specific G4S-sulfatase from *Z. galactanivorans* (ZGAL_3146, family S1_7) displays properties most consistent with *endo*-activity whereas the κ-carrageenan specific G4S-sulfatase S1_19B from *Pseudoalteromonas* is *exo*-acting on the non-reducing end. Finally, the DA2S-sulfatases from *Z. galactanivorans (*ZGAL_3151, family S1_17) and *Pseudoalteromonas* (S1_NC) are both *exo*-acting on non-reducing end DA2S residues; however, ZGAL_3151 is α-carrageenan specific whereas S1_NC has structural properties most consistent with action on both ι- and α-carrageenan. Thus, not only does the mode of oligosaccharide depolymerization differ between the two pathways but the order in which desulfation occurs must also differ[10].

Supplementary Methods.

Oligomeric state determination using size exclusion chromatography – Elution volumes (V_e) for protein molecular weight standards of 10 mg mL^{-1} albumin (MW: 66 kDa), 3 mg mL^{-1} conalbumin (MW: 75 kDa), 5 mg mL^{-1} alcohol dehydrogenase (MW: 150 kDa), and 4 mg mL^{-1} beta-amylase (MW: 200 kDa) were utilized to calibrate a HiPrep 16/60 Sephacryl S-300 HR column, 16 mm × 1600 mm (GE Healthcare). Blue dextran (MW: 2,000 kDa) was used to determine the void volume (V_o) and a standard curve was created plotting molecular mass *vs.* V_e/V_o for each respective protein standard. Samples of S1_19B and S1_NC at 10 mg mL^{-1} were applied to the column and their V_e/V_o values

plotted against the standard curve. All samples and standards were run at a flow rate of 0.5 mL/min in 500 mM NaCl and 20 mM Tris (pH 8.0).

Mass spectrometry – For liquid chromatography high resolution mass spectrometry/diode array detector/evaporative light scattering detector (LC-HRMS/DAD/ELSD), an Accela™ 1250 LC system was coupled to an Exactive™ mass spectrometer (Thermo Fisher Scientific) equipped with electrospray ionization source (HESI-II) probe. Through a flow-splitter, the LC eluent was simultaneously sent to a diode array detector (UltiMate 3000 DAD) to acquire UV signal and subsequently to an evaporative light scattering detector (Alltech 3300 ELSD). A makeup solution consisting of 0.1% formic acid in 80% methanol was delivered constantly at 100 µL to the MS. Separation was based on a modified method by Itoh et al. [11]. It was carried out on a Hypercarb column (100 x 2.1 mm, 5 µm Thermo Scientific) using mobile phase consisting of (A) 5 mM ammonium acetate pH 9.6 with 2% acetonitrile and (B) 5 mM ammonium acetate pH 9.6 with 80% acetonitrile, with a linear gradient from 5% B to 30% B in 30 min, and then to 100% B in another 5 min, before returning to initial gradients, at a flow-rate of 400 µL min^{-1}.

HRMS was acquired in negative polarity at 50,000 resolution. The following MS conditions were used: sheath flow 15, auxiliary gas flow rate 4; spray voltage -2.3 kV; both capillary and heater temperature at 250 °C. Mass range was scanned from m/z 100-2,000. MSMS was performed in high-energy collisional dissociation (HCD) scan at 10,000 resolution, using 60 eV. Maximum inject time for both MS and MSMS channels was at 50 ms.

Supplementary References.

1. Gobet, A. *et al.* Evolutionary Evidence of Algal Polysaccharide Degradation Acquisition by Pseudoalteromonas carrageenovora 9T to Adapt to Macroalgal Niches. *Front. Microbiol.* **9**, 2740 (2018).
2. Schultz-Johansen, M. *et al.* A Novel Enzyme Portfolio for Red Algal Polysaccharide Degradation in the Marine Bacterium Paraglaciecola hydrolytica S66T Encoded in a Sizeable Polysaccharide Utilization Locus. *Front. Microbiol.* **9**, 839 (2018).

3. McLean, M. W. & Williamson, F. B. kappa-Carrageenase from *Pseudomonas carrageenovora*. *Eur. J. Biochem.* **93**, 553–8 (1979).

4. Barbeyron, T., Henrissat, B. & Kloareg, B. The gene encoding the kappa-carrageenase of *Alteromonas carrageenovora* is related to beta-1,3-1,4-glucanases. *Gene* **139**, 105–9 (1994).

5. Michel, G. *et al.* The kappa-carrageenase of *P. carrageenovora* features a tunnel-shaped active site: a novel insight in the evolution of Clan-B glycoside hydrolases. *Structure* **9**, 513–25 (2001).

6. Matard-Mann, M. *et al.* Structural insights into marine carbohydrate degradation by family GH16 κ-carrageenases. *J. Biol. Chem.* **292**, 19919–19934 (2017).

7. Hettle, A. G. *et al.* The Molecular Basis of Polysaccharide Sulfatase Activity and a Nomenclature for Catalytic Subsites in this Class of Enzyme. *Structure* **26**, 747-758.e4 (2018).

8. Krissinel, E. & Henrick, K. Inference of macromolecular assemblies from crystalline state. *J. Mol. Biol.* **372**, 774–97 (2007).

9. Dierks, T., Lecca, M. R., Schlotterhose, P., Schmidt, B. & von Figura, K. Sequence determinants directing conversion of cysteine to formylglycine in eukaryotic sulfatases. *EMBO J.* **18**, 2084–91 (1999).

10. Ficko-Blean, E. *et al.* Carrageenan catabolism is encoded by a complex regulon in marine heterotrophic bacteria. *Nat. Commun.* **8**, 1685 (2017).

11. Itoh, S. *et al.* Simultaneous microanalysis of N-linked oligosaccharides in a glycoprotein using microbore graphitized carbon column liquid chromatography-mass spectrometry. *J. Chromatogr. A* **968**, 89–100 (2002).

12. Kelley, L. A. & Sternberg, M. J. E. Protein structure prediction on the Web: a case study using the Phyre server. *Nat. Protoc.* **4**, 363–71 (2009).

13. Robb, C. S., Reisky, L., Bornscheuer, U. T. & Hehemann, J.-H. Specificity and mechanism of carbohydrate demethylation by cytochrome P450 monooxygenases. *Biochem. J.* **475**, 3875–3886 (2018).

14. Yun, E. J., Yu, S. & Kim, K. H. Current knowledge on agarolytic enzymes and the industrial potential of agar-derived sugars. *Appl. Microbiol. Biotechnol.* **101**, 5581–5589 (2017).

15. Pluvinage, B., Hehemann, J. H. & Boraston, A. B. Substrate recognition and hydrolysis by a family 50 exo-β-agarase, aga50D, from the marine bacterium *Saccharophagus degradans*. *J. Biol. Chem.* **288**, 28078–28088 (2013).
16. Almagro Armenteros, J. J. *et al.* SignalP 5.0 improves signal peptide predictions using deep neural networks. *Nat. Biotechnol.* **37**, 420–423 (2019).
