# Supplementary material for: Insights into the κ/ι-carrageenan metabolism pathway of some marine Pseudoalteromonas species
Source: Commun Biol. 2019 Dec 19;2:474. doi: 10.1038/s42003-019-0721-y (PMC6923384; doi:10.1038/s42003-019-0721-y)
Supplement: Supplementary file 2 — Description of Additional Supplementary Files [file 42003_2019_721_MOESM2_ESM.pdf]

Description of Additional Supplementary Files

File Name: **Supplementary Data 1**
Description: This table contains the results of the RNAseq experiment.
