# Supplementary material for: Insights into the κ/ι-carrageenan metabolism pathway of some marine Pseudoalteromonas species
Source: Commun Biol. 2019 Dec 19;2:474. doi: 10.1038/s42003-019-0721-y (PMC6923384; doi:10.1038/s42003-019-0721-y)
Supplement: Supplementary file 5 — Reporting Summary [file 42003_2019_721_MOESM5_ESM.pdf]

nature research

Corresponding author(s): Alisdair B Boraston

Last updated by author(s): Sep 30, 2019

Reporting Summary

Nature Research wishes to improve the reproducibility of the work that we publish. This form provides structure for consistency and transparency in reporting. For further information on Nature Research policies, see Authors & Referees and the Editorial Policy Checklist.

Statistics

For all statistical analyses, confirm that the following items are present in the figure legend, table legend, main text, or Methods section.

n/a	Confirmed	
☐	☒	The exact sample size (*n*) for each experimental group/condition, given as a discrete number and unit of measurement
☐	☒	A statement on whether measurements were taken from distinct samples or whether the same sample was measured repeatedly
☒	☐	The statistical test(s) used AND whether they are one- or two-sided<br>*Only common tests should be described solely by name; describe more complex techniques in the Methods section.*
☒	☐	A description of all covariates tested
☒	☐	A description of any assumptions or corrections, such as tests of normality and adjustment for multiple comparisons
☒	☐	A full description of the statistical parameters including central tendency (e.g. means) or other basic estimates (e.g. regression coefficient) AND variation (e.g. standard deviation) or associated estimates of uncertainty (e.g. confidence intervals)
☒	☐	For null hypothesis testing, the test statistic (e.g. *F*, *t*, *r*) with confidence intervals, effect sizes, degrees of freedom and *P* value noted<br>*Give P values as exact values whenever suitable.*
☒	☐	For Bayesian analysis, information on the choice of priors and Markov chain Monte Carlo settings
☒	☐	For hierarchical and complex designs, identification of the appropriate level for tests and full reporting of outcomes
☒	☐	Estimates of effect sizes (e.g. Cohen's *d*, Pearson's *r*), indicating how they were calculated

Our web collection on statistics for biologists contains articles on many of the points above.

Software and code

Policy information about availability of computer code

Data collection	SoftMax Pro 6.2.1, HKL 3000, Blue-ice, Web-ice, MxDC
Data analysis	Excel (various versions), A5-miseq, SPAdes, Torrent Suite 5.10.1, Geneious Prime 2019.1.3, GraphPad Prism 5.0, CCP4/CCP4i 7.0.076, MOSFLM, SCALA, PHASER 2.7.17, BUCCANEER 1.6.3, COOT 0.8.9.2, REMAC5.9.0238, AutoSol, MOPROBITY, Phenix 1.13

For manuscripts utilizing custom algorithms or software that are central to the research but not yet described in published literature, software must be made available to editors/reviewers. We strongly encourage code deposition in a community repository (e.g. GitHub). See the Nature Research guidelines for submitting code & software for further information.

Data

Policy information about availability of data

All manuscripts must include a data availability statement. This statement should provide the following information, where applicable:

- Accession codes, unique identifiers, or web links for publicly available datasets
- A list of figures that have associated raw data
- A description of any restrictions on data availability

The coordinates and structure factors for all X-ray crystal structures have been deposited under the PDB IDs 6PNU, 6POP, 6PRM, 6PSM, 6PSO, 6PTR, 6PT6, 6PT9, 6PTK, and 6PTM. The P. fuliginea PS2, PS42, and PS47 Whole Genome Shotgun projects have been deposited at DDBJ/ENA/GenBank under the accessions SEUL00000000, SEUK00000000, and SEUJ00000000, respectively. The P. distincta U2A and Pseudoalteromonas sp. FUC4 Whole Genome Shotgun projects have been deposited under the accessions SEUH00000000 and SEUI00000000.

Field-specific reporting

Please select the one below that is the best fit for your research. If you are not sure, read the appropriate sections before making your selection.

☒ Life sciences ☐ Behavioural & social sciences ☐ Ecological, evolutionary & environmental sciences

For a reference copy of the document with all sections, see nature.com/documents/nr-reporting-summary-flat.pdf

Life sciences study design

All studies must disclose on these points even when the disclosure is negative.

Sample size	Standard replicate numbers for biochemical or microbiological assays of triplicate or quadruplicate were chosen.
Data exclusions	No data was excluded.
Replication	Where necessary, multiple biological replicates were used, as indicated in the manuscript. Results were reproducible.
Randomization	This is not relevant as there were no specific test groups.
Blinding	Blinding was not possible or relevant in biochemical assays.

Reporting for specific materials, systems and methods

We require information from authors about some types of materials, experimental systems and methods used in many studies. Here, indicate whether each material, system or method listed is relevant to your study. If you are not sure if a list item applies to your research, read the appropriate section before selecting a response.

Materials & experimental systems

n/a	Involved in the study	
☒	☐	Antibodies
☒	☐	Eukaryotic cell lines
☒	☐	Palaeontology
☒	☐	Animals and other organisms
☒	☐	Human research participants
☒	☐	Clinical data

Methods

n/a	Involved in the study	
☒	☐	ChIP-seq
☒	☐	Flow cytometry
☒	☐	MRI-based neuroimaging
